# Bioinspired exosome-SiO_2_ nanohybrid therapeutic for rheumatoid arthritis treatment

**DOI:** 10.7150/thno.108296

**Published:** 2025-05-30

**Authors:** Qicui Zhu, Ruofei Chen, Xueting Wu, Yuanyuan Zhou, Zexin Wang, Huaixuan Zhang, Haofang Zhu, Lingyun Sun, Zongwen Shuai

**Affiliations:** 1 Department of Rheumatology and Immunology, The First Affiliated Hospital of Anhui Medical University, Hefei, 230022, China; 2 Department of Rheumatology and Immunology, The Second Affiliated Hospital of Anhui Medical University, Hefei, 230601, China; 3 Inflammation and Immune Mediated Diseases Laboratory of Anhui Province, Hefei 230032, China; 4 Department of Rheumatology and Immunology, Institute of Translational Medicine, The Affiliated Drum Tower Hospital of Nanjing University Medical School, Nanjing, 210008, China

**Keywords:** stem cells, exosomes, methotrexate, nanocarrier, rheumatoid arthritis

## Abstract

**Objective:** Due to their anti-inflammatory and immunomodulatory capabilities, adipose-derived stem cells (ADSC) are currently considered a promising option for the management of rheumatoid arthritis (RA). To tackle the problems of immunogenicity and tumorigenicity linked to the direct use of cells, current research is focused on the development of effective nanomedicines utilizing ADSC-derived exosomes (ADSC-EXO) for cell-free regenerative medicine.

**Methods:** Methotrexate (MTX) was loaded into mesoporous silica through physical adsorption to produce SiO_2_-MTX, with subsequent incorporation into ADSC-EXO via ultrasonication to produce AE@SiO_2_-MTX. Particle size, surface charge, and stability were characterized using dynamic light scattering (DLS) and zeta potential analysis. *In vitro*, the effects of the nanomaterials were evaluated by assessing the inverse polarization effect of AE@SiO_2_-MTX on RAW264.7 macrophages, as well as on the migration and invasion capabilities of fibroblast-like synovial cells (FLS).* In vivo,* targeting and therapeutic effects on joint inflammation were examined using adjuvant-induced arthritis (AIA) and collagen-induced arthritis (CIA) mouse models.

**Results:** The AE@SiO₂-MTX demonstrated sustained drug release, high biocompatibility, and rapid cellular internalization. *In vitro*, the delivery system alleviated chronic inflammation by inducing macrophage polarization from the pro-inflammatory M1 to the anti-inflammatory M2 phenotype, as well as suppressing FLS migration and invasion. *In vivo* studies revealed that administration of ADSC-EXO outperformed ADSC transplantation in alleviating RA symptoms. Intravenously delivered AE@SiO₂-MTX exhibited targeted accumulation in inflamed joints, significantly reducing joint swelling, synovial hyperplasia, and bone/cartilage degradation in CIA model mice.

**Conclusions:** The findings show that AE@SiO₂-MTX is a robust cell-free therapeutic platform for RA management. Synergy between the immunomodulatory properties of ADSC-EXO and MTX controlled release, this system can overcome the limitations of conventional cell therapies and achieve targeted anti-inflammatory and tissue-protective effects. This strategy offers a promising translational avenue for RA treatment.

## Introduction

Rheumatoid arthritis (RA) is a chronic autoimmune disorder with widespread deleterious effects, including joint destruction, synovial hyperplasia, erosion of cartilage and bone, and damage beyond the joints 1-3. Activated macrophages and hyperproliferating fibroblast-like synovial cells (FLS) act as key drivers of RA progression. During the early stages of the disease, macrophage infiltration at inflammation sites triggers the hyperactivation and proliferation of FLS through the sustained release of pro-inflammatory cytokines 4. Concurrently, increased generation of reactive oxygen species (ROS) induces direct oxidative damage to the articular cartilage and bone matrices 5, while perpetuating a hypoxic microenvironment with upregulated expression of hypoxia-inducible factor-1α (HIF-1α) and vascular endothelial growth factor (VEGF), thereby exacerbating synovial hyperplasia and angiogenesis 6. Methotrexate (MTX) remains the first-line disease-modifying anti-rheumatic drug (DMARDs) for RA management 7-9. However, oral administration of MTX has several disadvantages, including the poor aqueous solubility, rapid metabolism, and non-specific tissue distribution of the drug. Systemic administration often leads to hematological complications, including anemia, leukopenia, and thrombocytopenia, as well as other adverse effects 10-13. To overcome these challenges, various drug delivery systems utilizing nano- and micro-particles have been developed. Previous research has demonstrated the effectiveness of nanoparticles in the specific delivery of therapeutic drugs to inflamed tissues 14-20; however, nanoparticles frequently show poor biocompatibility, limited drug loading efficiency, off-target distribution, and rapid metabolism within RA-affected joints. These issues result in reduced efficacy upon repeated administration and an increased risk of systemic side effects. Therefore, the development of nanodrug delivery vectors that can effectively target RA-affected joints and prolong the circulation time of MTX *in vivo* is crucial.

Exosomes represent an important subtype of extracellular vesicles with diameters ranging from 30 to 150 nm 21, 22. Exosomes are formed from lipid bilayers and transport a variety of bioactive molecules, including lipids, nucleic acids (DNA, mRNA, miRNA, and tRNA), and proteins 23. Immunologically active exosomes are produced by various cell types, including macrophages and tumor cells, and retain the functional characteristics of their parent cells 24. Although exosomes derived from M2 macrophages have shown promise as anti-inflammatory nanocarriers for RA therapy 25, 26, mesenchymal stem cell-derived exosomes (MSC-EXO) offer distinct advantages. MSC-EXO exhibit superior therapeutic versatility, due to their dual immunomodulatory capacity (balancing pro- and anti-inflammatory responses), low immunogenicity, robust tissue regenerative potential, and ability to target inflammatory tissues conferred by surface markers, such as CD29, CD44, and CD73 27-29. These attributes underlie their demonstrated therapeutic efficacy in a variety of applications, including bone regeneration 30, myocardial repair 31, neurodegenerative diseases management 32, and immune disorder modulation 33. Notably, exosomes isolated from olfactory 34, gingival 35, and umbilical cord 36 mesenchymal stem cells (MSC) have shown multifaceted therapeutic actions in RA, simultaneously mitigating synovial inflammation and halting joint destruction. However, the therapeutic potential of adipose-derived stem cell-derived exosomes (ADSC-EXO) as drug carriers for the treatment of RA has yet to be fully investigated.

**Figure 1:**
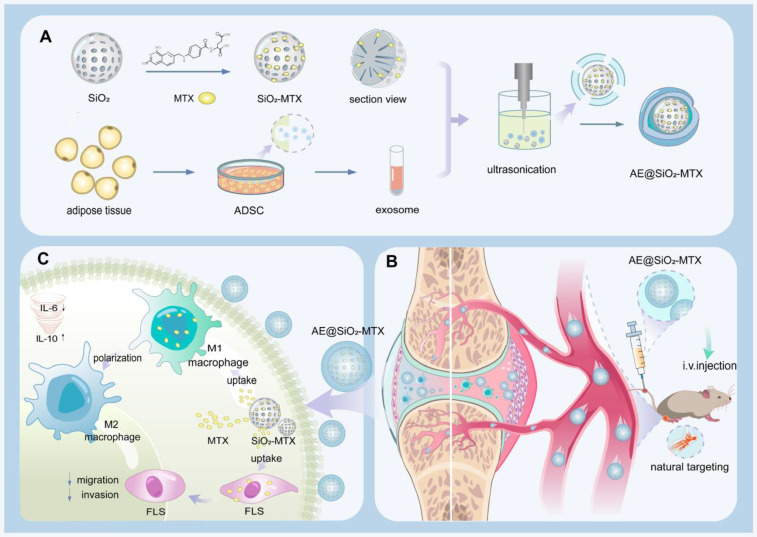
Schematic representation of the hierarchical nano-delivery system incorporating ADSC-EXO and MTX-loaded SiO_2_ nanoparticles for the treatment of RA. (A) Construction of AE@SiO_2_-MTX. (B) AE@SiO_2_-MTX specifically accumulated in inflamed joints via intravenous injection. (C) AE@SiO_2_-MTX alleviated RA symptoms by reversing the inflammatory phenotypes of macrophages and reduce the migration and invasion of FLS.

Metal nanoparticles have emerged as promising candidates for drug delivery systems due to their exceptional physicochemical properties and demonstrated biosafety 37. Specifically, silica nanoparticles (SiO₂) have garnered significant attention in pharmaceutical applications, due to their unique advantages such as high surface-to-volume ratios, tunable mesoporous structures, outstanding biological compatibility, and controlled release capabilities that enable prolonged therapeutic effects 38, 39. Thus, it is possible that the combination of ADSC-EXO and SiO_2_ as a hierarchical MTX carrier may represent a novel means of managing RA. In this study, we encapsulated negatively charged MTX within positively charged SiO_2_ through electrostatic interactions, and subsequently incorporated this complex into the ADSC-EXO *via* ultrasonication to form AE@SiO_2_-MTX (Figure 1). By adjusting the feeding ratio of MTX, we fabricated AE@SiO_2_-MTX with optimal drug-loading efficiency, resulting in both stable particle sizes and zeta potentials. Compared to free MTX, AE@SiO_2_-MTX showed several advantages, including sustained drug release capability, excellent biocompatibility, and rapid *in vitro* uptake. AE@SiO_2_-MTX also induced polarization of macrophages toward an anti-inflammatory phenotype, significantly reduced ROS production, and suppressed FLS migration and invasion *in vitro*. Notably, AE@SiO_2_-MTX could specifically target inflamed joints following intravenous injection, exhibiting promising therapeutic effects by reducing swellings, bone destruction, and cartilage damage in collagen-induced arthritis (CIA) model mice. These findings emphasize the significant therapeutic potential of this hierarchical MTX carrier in the treatment of RA and other autoimmune disorders.

## Materials and methods

### Materials

MTX was purchased from Solarbio Science & Technology Co., Ltd (Beijing, China). Mesoporous silica (50 nm), Cy5-SiO_2_ (50 nm), Cy5-MTX and FITC-MTX were purchased from Ruixi Biotechnology Co., LTD (Xian, China). 4',6-Diamidino-2-phenylindole (DAPI), the cell counting kit-8 (CCK-8), the calcein AM and propidium iodide (PI) staining kit, 2',7'-dichlorodihydrofluorescein diacetate (DCFH-DA) were obtained from Yeasen Biotechnology Co., Ltd. (Shanghai, China). Exosomes extraction kit purchased from TransGen Biotech (Beijing, China). Dulbecco's-modified Eagle medium and foetal bovine serum (FBS) were purchased from United States Origin, CLARK. Immunization-grade bovine type II collagen solution (20022, Chondrex), Complete Freund's Adjuvant (7001 and 7027, Chondrex), and Incomplete Freund's Adjuvant (7002, Chondrex) were obtained from Chondrex (USA). Elabscience Biotechnology Co., Ltd. supplied the Mouse IL-6 and IL-10 enzyme-linked immunosorbent assay (ELISA) kits. Corning provided the matrix. Lipopolysaccharide (LPS) and DiR dye were purchased from MedChemExpress (MCE, USA). DiD fluorescent dye was acquired from Aladdin Biochemical Technology Co., Ltd (Shanghai, China). Type I collagenase was procured from Sigma-Aldrich (St. Louis, MO, USA). Primary antibodies including MMP3, TNF-α, CD68, iNOS, and CD206 were purchased from Proteintech (Rosemont, IL, USA), while antibodies against MMP13, IL-6, CD81, TSG101 and Calnexin were obtained from Boster Biological Technology (Wuhan, China). Flow cytometry antibodies for CD86 and CD206 were sourced from BioLegend.

### Cell culture

The RAW 264.7 macrophages were obtained from IMMOCELL (Xiamen, Fujian, China) and FLS were donated by the Rheumatology Laboratory of Nanjing Medical University. ADSC were derived from adipose tissue obtained from patients who had provided written informed consent prior to surgery. For ADSC isolation, adipose tissues were digested with 2 mg/mL collagenase type I for 1 h at 37 °C, followed by centrifugation at 300 × g for 10 min. All procedures were approved by the Ethics Committee of Anhui Medical University (Hefei, China; approval no. LLSC20230847) and conducted in accordance with recognized ethical guidelines. Cells from the stromal vascular fraction were collected and passed through a 100 μm porous membrane (Cell Strainer, BKMAMLAB) for filtration. ADSC were seeded at a starting density of 4000 cells/cm² in DMEM-F12, enriched with 10% FBS and 1% penicillin. At 80% confluence, the medium was replaced with serum-free F12 medium**,** and after 48 h of incubation, the conditioned medium was collected as the ADSC supernatant. FLS and RAW 264.7 macrophages were maintained in DMEM/F12 medium (Gibco, Germany) and DMEM medium (Gibco, Germany), respectively, both supplemented with 10% FBS and 1% penicillin/streptomycin, and cultured at 37 °C in a 5% CO₂ atmosphere. Cells were harvested at the logarithmic growth phase for subsequent experiments, including passaging and stimulation. Cell counting was performed using a hemocytometer.

### ADSC-EXO purification and characterization

To acquire ADSC-EXO, ADSC were seeded at a density of 10,000 cells per well, grown to 70-80% confluence, and incubated for 48 h in serum-free medium. Using ultracentrifugation on a QPTima MAX-XP Ultra-High system (Beckman Coulter, USA), exosomes were isolated from the conditioned medium of ADSC as described. Briefly, the cell culture supernatant was centrifuged at 300 × g for to remove the cells, then the supernatant was centrifuged again at 2,000 × g for 10 minutes to remove the dead cells, and finally the supernatant was centrifuged at 10,000 × g for 30 minutes to further remove cellular debris. After obtaining the supernatant, it was ultracentrifuged at 100,000 × g for 70 minutes to precipitate exosomes. The exosomes pellets were then washed with a large volume of PBS and centrifuged at 100,000 × g for another 70 minutes to remove impurities. The exosomes extraction kit was also employed to isolate exosomes. In summary, the cell culture supernatant underwent centrifugation at 3000 × g for 30 minutes to remove cellular debris. Afterward, the exosomes extraction reagent was introduced, and the mixture was inverted. It was then left to settle overnight at 4 °C, followed by a final centrifugation at 10000 × g for 30 minutes to collect the purified exosomes. After purification, exosomes were resuspended in PBS and stored at -80 °C, with concentrations measured using the BCA kit. All processes were conducted at 4 °C under sterile conditions.

The exosomes were diluted to a concentration of 1 mg/mL, and the size distribution of the purified exosomes was measured using dynamic light scattering (DLS; NS-90Z, OMEC, China). The nanoscale morphology of exosomes was analyzed by transmission electron microscopy (TEM; Talos L120C G2, Thermo Fisher Scientific, USA). Exosomes markers including CD81, TSG101, and Calnexin were detected by western blot analysis. To achieve triple fluorescence labeling, the ADSC membrane was labeled with DiR fluorescent dye, with FITC-MTX and Cy5-SiO_2_ serving as the core. Cellular observation and image acquisition were performed using confocal laser scanning microscopy (CLSM; ZEISS LSM900).

For western blot analysis, ADSC and exosomes were lysed using RIPA lysis buffer. Subsequently, the lysates were boiled for 5 min and centrifuged at 12,000 × g for 15 min at 4 °C, followed by electrophoretic separation of the supernatant on 10% SDS-PAGE gels. The proteins were then transferred to a PVDF membrane using standard wet transfer methods for immunoblotting with primary antibodies against CD81 (1: 1000), TSG101 (1: 1000), and Calnexin (1: 1000). After washing, the membrane was incubated with horseradish peroxidase-conjugated goat anti-rabbit IgG secondary antibody (1: 5000) for 1 h at room temperature. The immunoreactive bands were detected with enhanced chemiluminescence reagent using a ChemiDoc MP imaging system.

### Synthesis of SiO_2_-MTX

To synthesize SiO_2_-MTX, 2 mg SiO_2_ mixed into 4 ml MTX solution (0.25 mg/mL) and stirred slowly for 24 h at room temperature. Subsequently, the mixture was centrifuged at 13000 rpm to collect SiO_2_-MTX. The final product was washed with deionized water two times and stored at 4 ℃ for further use.

### Preparation of AE-MTX and AE@SiO_2_-MTX

To encapsulate SiO_2_-MTX into ADSC-EXO, 1 mg of ADSC-EXO and 2.5 mg of SiO_2_-MTX were combined with in PBS, followed by sonication using an ultrasonic homogenizer (Xiaomei, China) at 20% amplitude for 6 cycles (30 seconds on/30 s off per cycle, 3 minutes total duration) with 2 minutes cooling intervals between cycles. Free MTX was eliminated using an ultrafiltration tube, then the solution was then incubated at 37 °C for 1 h to restore the exosome membrane. The same method was applied to synthesize AE-MTX. The final MTX concentrations in both formulations were verified by ultraviolet-visible spectroscopy (UV-Vis) at 303 nm.

### Characterization of AE@SiO_2_-MTX

The morphology of AE@SiO₂-MTX was observed using TEM after negative staining with 3% phosphotungstic acid. DLS was used to measure the size distribution and zeta potential of both ADSC-EXO and AE@SiO₂-MTX. The encapsulation efficiency (EE) and drug loading capacity (DLC) of MTX were assessed by collecting AE@SiO_2_-MTX via centrifugation at 3000 × g for 5 minutes and dissolving it in pure water. The amount of MTX in the supernatant of AE@SiO_2_-MTX was measured by the UV-vis absorption (TECAN, Austria GmbH). The EE and DLC were calculated using the following formulas:

EE (%) = MTX encapsulated in AE@SiO_2_-MTX / Total MTX× 100%.

DLC (%) = MTX encapsulated in AE@SiO_2_-MTX / Weight of AE@SiO_2_-MTX × 100%.

To assess the stability of AE@SiO₂-MTX, the particle size distribution was monitored over 7 days at 37 °C using DLS. To simulate physiological conditions, the size and zeta potential changes of AE@SiO₂-MTX in 50% FBS were evaluated. For drug release studies, 1 mg MTX-loaded AE@SiO₂-MTX and AE-MTX nanoparticles were suspended in 30 mL PBS (pH 7.4) and placed in dialysis bags (Mw = 1 kDa) at 37 °C. At predetermined time intervals (0.5, 1, 2, 4, 6, 8, 24, 48, and 72 h), 1 mL aliquots were collected by centrifugation (with triplicate samples per time point) and replaced with fresh PBS. AE-MTX served as the control group. The MTX concentration was quantified by UV-Vis absorption spectrometry, and cumulative release curves were generated following the protocol of manufacturer.

### *In Vitro* cellular vitality and uptake

To evaluate the cytotoxicity of ADSCs, EXO, MTX, AE-MTX, and AE@SiO₂-MTX on RAW 264.7 macrophages, cells (1×10⁴ cells/well) were seeded in 96-well plates and cultured for 24 h. Subsequently, the following treatments were added: ADSC-conditioned medium (200 μL/mL), EXO (1 μg/mL), MTX, AE-MTX, or AE@SiO₂-MTX (all at 0.5 μg/mL MTX-equivalent concentration). After 24, 48, and 72 h of incubation, the supernatant was removed and replaced with fresh medium containing 10% CCK-8 solution (100 μL/well), followed by incubation at 37 °C for 1 h. The absorbance at 450 nm was measured using a microplate reader. For live/dead cell assessment, samples were cultured in 6-well plates overnight, then treated with ADSC-conditioned medium (200 μL/mL), EXO (1 μg/mL), MTX, AE-MTX, and AE@SiO₂-MTX (0.5 μg/mL MTX-equivalent). After 24 h incubation, cells were stained with 500 μL of Calcein-AM/PI working solution for 20 min and immediately imaged using a fluorescence microscope (Nikon Ti2-A, Japan).

The cytotoxicity assessment of FLS was performed using an identical protocol, with the following specific conditions: FLS were seeded in 96-well plates at a density of 0.5×10⁴ cells/well and treated with ADSC-conditioned medium (200 μL/mL), EXO (2 μg/mL), MTX, AE-MTX, and AE@SiO₂-MTX (all at 1 μg/mL MTX-equivalent concentration). Cell viability was assessed using the previously described CCK-8 assay and live/dead staining protocols.

To obtain activated macrophages, RAW 264.7 macrophages were stimulated with 10 μg/mL LPS for 24 h. Both LPS-activated and non-activated macrophages were seeded in 6-well plates and cultured overnight. Subsequently, FITC-labeled MTX and AE@SiO₂-MTX (0.5 μg/mL MTX-equivalent) were co-cultured with the macrophages for 12 h. The cellular uptake efficiency was evaluated using CLSM and flow cytometry (Beckman CytoFLEX, USA).

The cellular uptake experiments in FLS were performed following the same protocol. Briefly, FLS were seeded in 6-well plates and incubated with FITC-labeled MTX and AE@SiO₂-MTX (1 μg/mL MTX-equivalent). Quantitative and qualitative uptake analysis were performed after 2 h and 8 h incubation periods using both CLSM and flow cytometry.

### ROS detection by fluorescence microscopy

ROS generation in macrophages was assessed using DCFH-DA staining. Briefly, LPS-stimulated RAW 264.7 cells cultured in 6-well plates were incubated for 24 h with PBS, ADSC-conditioned medium (200 μL/mL), EXO (1 μg/mL), MTX, AE-MTX, AE@SiO₂-MTX (all at 0.5 μg/mL MTX-equivalent concentration). Following treatment, cells were incubated with 10 μM DCFH-DA for 30 min at 37 °C, thoroughly washed with PBS, and immediately analyzed using an inverted fluorescence microscope (excitation/emission: 488/525 nm). Fluorescence intensity was quantified from three random fields per well using Image J software.

### Phenotypic transformation of RAW 264.7 macrophages

To investigate macrophage phenotypic transformation, RAW 264.7 macrophages were seeded in 6-well plates and stimulated with 10 μg/mL LPS for 24 h. Following activation, cells were treated for 24 h with PBS (control), ADSC-conditioned medium (200 μL/mL), EXO (1 μg/mL), MTX, AE-MTX, or AE@SiO₂-MTX (all at 0.5 μg/mL MTX-equivalent), then fixed with 4% paraformaldehyde. Fixed cells were incubated overnight at 4 °C with primary antibodies against CD68 (pan-macrophage marker), inducible nitric-oxide synthase (iNOS, M1 marker), and CD206 (M2 marker), followed by 2 h incubation with species-matched fluorescent secondary antibodies and nuclear staining with DAPI (1 μg/mL). Immunofluorescence images were acquired using CLSM. For flow cytometric analysis, cells were stained with FITC-conjugated anti-CD86 (M1 marker) and APC-conjugated anti-CD206 (M2 marker) (BioLegend) at 4 °C for 40 min, then analyzed by flow cytometry to quantify macrophage polarization phenotypes.

### *In vitro* chemotaxis assay

The migration and invasion capacities of FLS were evaluated *in vitro* assays. For transwell migration analysis, FLS (2×10⁴ cells/well) were seeded in the upper chamber of an 8 μm pore transwell system (Corning), while the lower chamber contained medium with 20% FBS as chemoattractant, along with PBS, ADSC-conditioned medium (200 μL/mL), EXO (2 μg/mL), MTX, AE-MTX, or AE@SiO₂-MTX (all at 1 μg/mL MTX-equivalent). After 24 h co-culture, non-migrated cells were removed, and migrated cells on the lower membrane surface were fixed and stained with 0.1% crystal violet for microscopic quantification. For invasion assessment, chambers were pre-coated with diluted Matrigel (1:80 in serum-free medium) before cell seeding. In parallel, wound healing assays were performed by creating uniform scratches in confluent FLS monolayers in 6-well plates using a 200 μL pipette tip, followed by treatment with the same experimental conditions. After removing cellular debris with PBS, cell migration was monitored at 0 h and 12 h post-scratching using live-cell imaging with Calcein-AM staining (2 μM). Migration areas were quantified from triplicate wells using ImageJ software.

### RNA sequencing and bioinformatics analysis

Total RNA was extracted from FLS, including the negative control (NC) group and the AE@SiO₂-MTX treated group, using TRIzol reagent. The purity and integrity of the RNA were assessed by measuring the A260/A280 ratio. Subsequently, an RNA sequencing library was constructed and sequenced on the Illumina NovaSeq 6000 platform with 150 bp paired-end reads. Differentially expressed genes (DEGs) were identified using the DESeq2 software package. To further elucidate the biological processes associated with cell migration and invasion, functional enrichment analysis, including Gene Ontology (GO) and Kyoto Encyclopedia of Genes and Genomes (KEGG) pathway analysis, were conducted on the selected DEGs. Finally, the expression levels of key candidate genes were validated using reverse transcription quantitative polymerase chain reaction (RT-qPCR).

### AIA model induction and treatment

This study was approved by the Ethics Committee. Male Sprague-Dawley (SD) rats, aged 6-8 weeks and weighing approximately 300 g, were housed in a specific pathogen-free (SPF) environment maintained at a temperature of 23 °C. Following one-week acclimatization period, an adjuvant-induced arthritis (AIA) mouse model was successfully established via subcutaneous injection of 100 μL of complete Freund's adjuvant (CFA, 7027) into the right hind paw pad. Subsequently, the rats were randomly divided into four groups (n = 3 per group): control group, AIA group (500 μL PBS), ADSC group (5 × 10^5^ cells) and EXO group (2.5 mg/kg). The therapeutic drug was administered via the tail vein every three days for four times. During the experiments, changes in paw volume and arthritis score were monitored at regular intervals. Following the completion, infrared thermal imaging technology (Hikon P09) was employed to detect temperature in the ankle joints. X-ray imaging system (VetiX S32) was employed to assess bone destruction, while HE staining as well as saffranine O staining were utilized for the pathological examination of ankle tissues.

### CIA model induction and treatments

Male DBA/1 mice, aged 6-8 weeks and weighing between 20-30 g, were obtained from Jiangsu Jicuiyaokang Biotechnology Co., LTD. The animal experiments were carried out in a pathogen-free environment, complying with the Ethics Committee approved guidelines for the care and use of laboratory animals. The mice were kept in standard stainless-steel cages at a temperature of 23 °C. Prior to the experiment, they went through an acclimation phase with access to a standard diet and water for the entire study. The CIA mouse model was created by intradermally injecting bovine type II collagen along with Complete Freund adjuvant in a 1:1 ratio. On the 21st day, an emulsion of bovine type II collagen and Incomplete Freund adjuvant in a 1:1 ratio was injected to enhance the molding rate.

A biological distribution test was performed using an arthritis model, with all mice split into four groups. MTX group comprised of mice in the CIA model group that received Cy5-MTX, while the other groups consisted of mice in the CIA model that received the membrane fluorescent dye DiD. All groups administered a standardized concentration of Cy5-MTX (5 μg/mL) and DiD (5 μg/mL) through tail intravenous injection. The IVIS imaging system was utilized to capture *in vivo* images of mice at designated time points (4 h and 24 h post-injection). For organ distribution analysis, mice were euthanized after 4 h and 24 h of treatments, followed by fluorescence imaging analysis on major tissue such as joints, hearts, livers, spleens, lungs and kidneys.

For histological analysis, paw tissues collected at 4 h and 24 h were embedded in OCT compound, snap-frozen in liquid nitrogen, and sectioned (20 μm thickness). Sections were immunostained with anti-F4/80 (1:1000) and anti-Vimentin (1:1000;) followed by species-matched fluorescent secondary antibodies, with cellular localization of fluorescence signals analyzed by CLSM.

Following successful CIA induction, the mice were randomly separated into five groups (n = 3 per group) and intravenously administered with PBS, EXO (2.5 mg/kg), MTX, AE-MTX, AE@SiO_2_-MTX (equivalent to MTX 2.5 mg/kg for 4 weeks) every seven days. The severity of RA in the treated mice were assessed by scoring their paws on a scale of 0-4 every week. The swelling of the hind paws was measured by using a volumeter at the same frequency. Upon completion of the treatment period, euthanized mice underwent comprehensive analysis. Limbs were fixed in 4% paraformaldehyde and analyzed by micro-CT (Sky Scan, Bruker; 50 kV, 500 μA, 65 ms exposure) with 3D reconstruction using CTAn software (Bruker) to quantify subchondral bone mineral density (BMD). Limb histology was analyzed using HE and safranin O. The criteria used for evaluating HE staining images of different joints included: 0 = normal synovial membrane; 1 = thickening of the synovial membrane and infiltration of cells; 2 = formation of masses and erosion of cartilage; 3 = erosion affecting both cartilage and subchondral bone; 4 = joint dysfunction and stiffness. Each metric was repeated twice to obtain an average score. Knee joint sections were immunostained with primary antibodies against TNF-α, IL-6, MMP3, and MMP13 (all 1: 200 dilution) followed by fluorescent secondary antibodies and DAPI counterstaining, with imaging performed by CLSM. Orbital blood samples were processed to obtain cell-free serum (3000 × g, 15 min) for ELISA quantification of IL-6/IL-10 and catalase activity measurements using commercial kits.

### Statistical analysis

Results are shown as averages and standard deviations (SD). Data analysis was conducted using Prism 9.0 Software (GraphPad). *T*-test for two independent samples, along with one-way and two-way analysis of variance (ANOVA) were performed. Statistically significant differences were denoted as *P < 0.05, **P < 0.01, ***P < 0.001; ****P < 0.0001, ns: no significance.

## Results and Discussion

To create the drug loading platform, electrostatic interactions were used to encapsulate MTX in SiO_2_, with adjustment of SiO_2_ to MTX ratios to 5:1, 2:1, and 1:1. The 2:1 ratio was found to induce appropriate encapsulation efficiency and drug-loading capacity, while also resulting in a sustained-release profile without an initial burst release effect. Therefore, this ratio was selected as the optimal formulation for use as the encapsulation material in subsequent experiments (Table S1). SiO_2_-MTX was incorporated into ADSC-EXO using ultrasonication, forming AE@SiO_2_-MTX with a stable zeta potential and uniform particle size.

**Figure 2:**
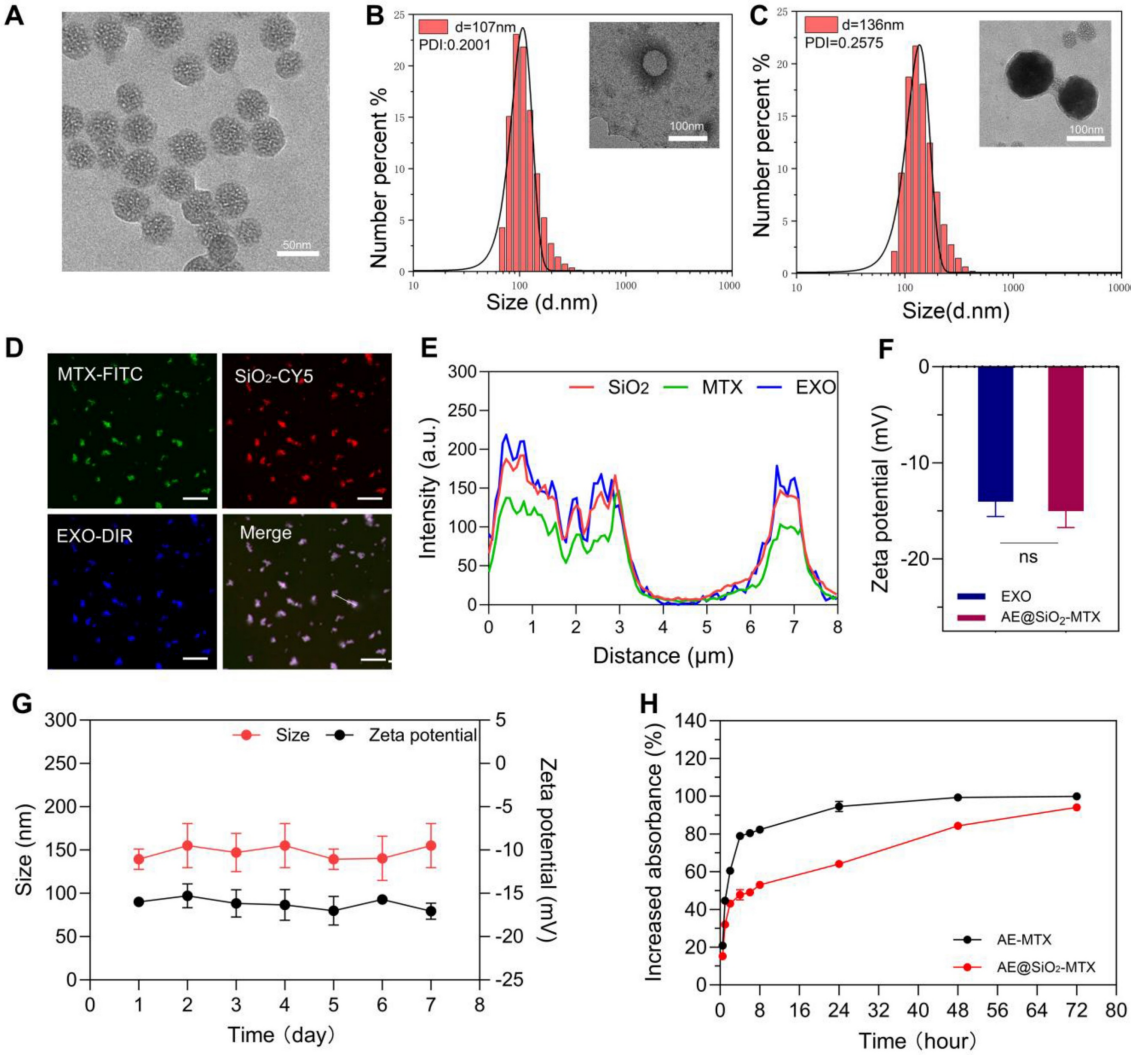
** The characterization of ADSC-EXO and AE@SiO_2_-MTX.** (A) TEM images of SiO_2_. (B-C) DLS analysis and TEM images of ADSC-EXO and AE@SiO_2_-MTX. (D-E) CLSM and line profile analysis of co-localization of AE@SiO_2_-MTX. Scale bar: 10 μm. (F) Zeta potential analysis of ADSC-EXO and AE@SiO_2_-MTX. (G) The stability of AE@SiO_2_-MTX in size and zeta potential, n = 3. (H) Release profile of MTX encapsulated in AE-MTX and AE@SiO_2_-MTX, n = 3. Data were expressed as mean ± SD, ns: no significance.

TEM revealed that the SiO_2_ nanoparticles were uniform in size with an average diameter of 50 nm (Figure 2A). DLS analysis indicated that the average particle size of the ADSC-EXO was 107 nm, with a polydispersity index (PDI) of 0.2001. Consistently, TEM evaluation showed that the ADSC-EXO appeared as circular nanovesicles enclosed within a membrane, with an approximate diameter of 100 nm (Figure 2B). Proteins specific for the ADSC-EXO, such as TSG101, CD81 and Calnexin, were identified using western blotting (Figure S1).

After coating with exosomes membranes from ADSC, the diameter of the resulting AE@SiO_2_-MTX was increased by approximately 80 nm. TEM examination revealed a distinct core-shell monodispersed nanostructure, confirming the successful coating of the ADSC-EXO membrane onto the surface of SiO_2_-MTX (Figure 2C). Consistent with these findings, the co-localized nanomaterials were visualized using CLSM following fluorescence labeling (Figure 2D). The co-expression and co-localization of FITC-MTX, Cy5-SiO_2_, and DiR-EXO were further verified using line profile analysis (Figure 2E). Additionally, AE@SiO_2_-MTX exhibited zeta potentials comparable to those of exosomes, with an average charge of approximately -15 mV (Figure 2F). These evaluations demonstrated the successful synthesis of AE@SiO_2_-MTX.

The stability of nanomaterials represents a fundamental prerequisite for their effectiveness. *In vitro*, at 37 °C and pH 7.4, the synthesized AE@SiO_2_-MTX nanoparticles exhibited minimal changes in zeta potential and increased slightly in size over a 7-day period (Figure 2G). This may likely be attributed to the aggregation and fusion effects induced by the phospholipid and cholesterol components on the surfaces of the exosome membranes. Additionally, throughout the entire observation period, the material maintained a consistently relatively low polydispersity index (PDI) (Figure S2A). To simulate the conditions of the *in vivo* microenvironment, we systematically investigated the variations in particle size and zeta potential characteristics of AE@SiO_2_-MTX in a system containing 50% FBS. It was found that over 24 h, the AE@SiO_2_-MTX nanoparticles maintained a stable particle size distribution and zeta potential values, providing critical evidence for their *in vivo* efficacy (Figure S2B). At 37 °C and pH 7.4, the release of MTX from AE@SiO_2_-MTX *in vitro* was significantly slower and more sustained compared to MTX release from AE-MTX (Figure 2H). Within the first 2 h, only 40% of MTX was released from AE@SiO_2_-MTX, while 60% of the MTX was released from AE-MTX. By 8 h, the rates of release were 50% and 80%, respectively. More than 90% of the drug in AE-MTX was released within 24 h, while MTX release from AE@SiO_2_-MTX occurred gradually over approximately 72 h. This was due to the strong adsorption capacity of SiO_2_ which provided adequate protection, thereby slowing drug release.

Macrophages are central to the immunopathogenesis of RA, acting as both triggers of inflammation and mediators of immune dysfunction and joint damage. Specifically, macrophages are pivotal in driving the pathological progression of RA through their production of pro-inflammatory cytokines, activation of the adaptive immune system, promotion of synovial tissue proliferation, and direct involvement in the degradation of cartilage and the bone matrix 40. Prior to functional evaluation, PBS, ADSC (supernatant 200 μL/mL), EXO (1 μg/mL), MTX, AE-MTX, and AE@SiO_2_-MTX (at an equivalent of 0.5 µg/mL) were co-cultured with RAW 264.7 macrophages to assess their cytocompatibility, measuring cell viability with CCK-8 assays. The findings showed that AE@SiO_2_-MTX exhibited minimal cytotoxicity during the tested time (Figure S3). As a further measure of cytotoxicity, calcein AM and PI, which emit green and red fluorescence, respectively, were used as co-stains to assess the viability of RAW 264.7 cells (Figure S4). The findings showed that macrophages treated with MTX and AE-MTX had the lowest viability compared to the other groups, aligning with the CCK-8 assay results. In contrast, RAW 264.7 cells exposed to AE@SiO_2_-MTX (0.5 µg/mL) showed similar rates of cell death at 24, 48, and 72 h to those observed in the ADSC and EXO groups, demonstrating its excellent biocompatibility. These findings indicate that AE@SiO_2_-MTX had the potential to serve as a nanodrug delivery system for mitigating the cytotoxic impact of MTX.

**Figure 3:**
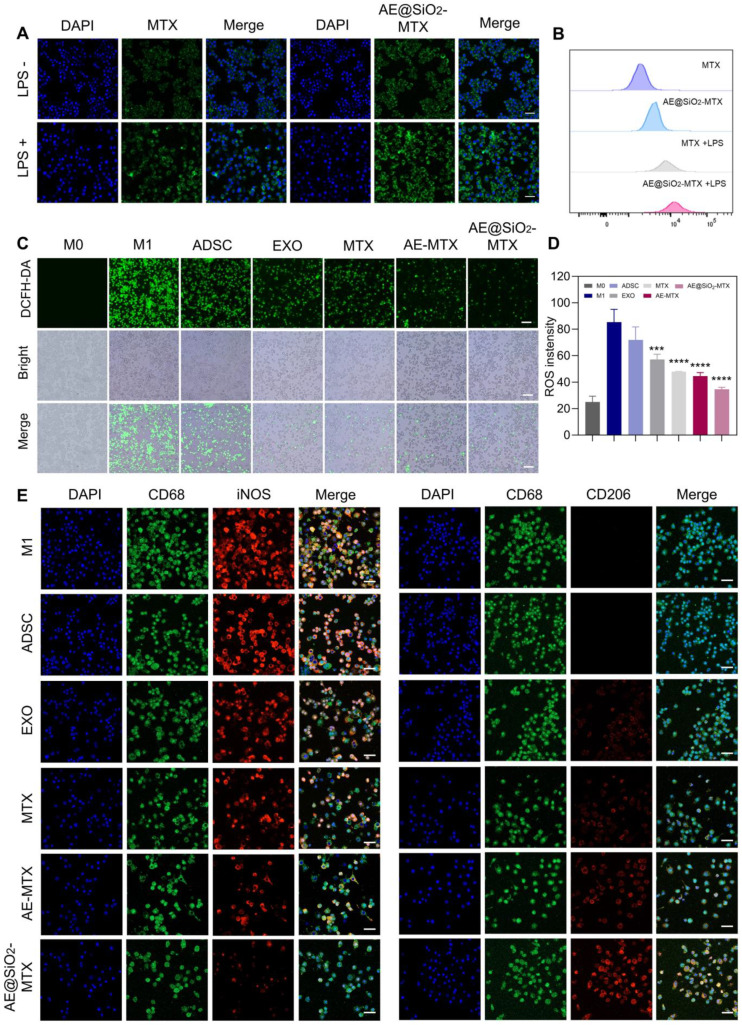
** AE@SiO_2_-MTX facilitated the transition of RAW 264.7 cells from M1 to M2 phenotype *in vitro*.** (A-B) Free FITC-MTX and FITC-labeled AE@SiO_2_-MTX uptake by M0 macrophages and M1 macrophages in CLSM and flow cytometer. Scale bar: 20 μm. (C) Representative fluorescence images and (D) quantitative analysis of ROS levels in M1 macrophages under different treatments. Scale bar: 100 μm. (E) Markers M0 (CD68, green), M1 (iNOS, red) and M2 (CD206, red) applied in immunofluorescence staining to examine M1 macrophage phenotype changes. Scale bar: 20 μm. Results were expressed as mean ± SD and One-way ANOVA was employed to evaluate statistical significance. ***P < 0.001, ****P < 0.0001.

Evaluation of the absorption of nanoparticles by activated macrophages is crucial for determining their effectiveness in RA. We performed uptake assays using M0 and M1 macrophages activated with LPS. Compared with the M0 macrophages, the M1 macrophages exhibited significantly enhanced uptake of FITC-labeled MTX. Activated macrophages demonstrated the highest amounts green fluorescence signal following exposure to FITC-labeled AE@SiO_2_-MTX. In contrast, macrophages not stimulated by LPS displayed markedly weaker green fluorescence signals (Figure 3A). Flow cytometry confirmed the CLSM images (Figure 3B). Specifically, flow cytometry indicated that AE@SiO_2_-MTX exhibited increased uptake in target cells, particularly in LPS-stimulated RAW264.7 cells. The enhanced uptake by M1 macrophages may be attributed to multiple coordinated cellular adaptations: (1) The upregulation of specific surface receptors and solute carriers, facilitating active transport 41; (2) Glycolytic metabolic reprogramming for adenosine triphosphate generation, providing sufficient energy to support receptor-mediated endocytosis and active transport processes 42; (3) Remodeling of membrane lipids, thus enhancing membrane fluidity and vesicle transport efficiency 43. The mechanism of cellular uptake was found to differ substantially between MTX and AE@SiO_2_-MTX. MTX uptake was entirely dependent on active transport mediated by folic acid carriers 44, while exosomes utilize a variety of uptake pathways, including (i) entosis, (ii) receptor-mediated endocytosis, and (iii) direct fusion with the plasma membrane 45, 46. In this study, the AE@SiO_2_-MTX nanoparticles were found to use the natural uptake pathways of exosomes. Compared to free MTX, this biomimetic surface modification markedly enhanced the intracellular internalization efficiency in M1 macrophages.

Given the pivotal role of ROS in RA inflammation, the development of strategies to effectively modulate ROS levels is of great significance. In this study, the fluorescent probe DCFH-DA was employed to evaluate the effects of various treatments on ROS levels. The results demonstrated that LPS-stimulated macrophages exhibited intense green fluorescence, indicating high levels of ROS and the successful induction of a high-ROS environment. Treatment with AE@SiO_2_-MTX reduced ROS levels significantly (Figure 3C-D). These findings unequivocally suggested that AE@SiO_2_-MTX possessed robust intracellular ROS-scavenging capability, which may be critical in alleviation of the inflammatory responses.

During RA development, M1 macrophages are responsible for producing inflammatory cytokines that promote RA progression, while M2 macrophages secrete anti-inflammatory factors. Altering macrophage polarization from the M1 to the M2 phenotype, determined using the markers iNOS for M1 and CD206 for M2, is seen as a promising treatment strategy. In this study, we investigated whether AE@SiO₂-MTX nanoparticles could also induce M2 polarization by reducing the proportion of M1-phenotype RAW264.7 cells. Immunofluorescence labeling demonstrated that AE@SiO₂-MTX significantly downregulated iNOS levels while upregulating those of CD206 in RAW264.7 cells (Figure 3E). Flow cytometry analysis (Figure S5) revealed a significantly higher proportion of CD86^+^ cells in the LPS-stimulated group (M1 group), confirming that LPS effectively induced M1-type polarization in RAW264.7 cells. In comparison, the AE@SiO₂-MTX nanoparticle-treated group exhibited markedly reduced numbers of CD86^+^ cells compared to the control group, demonstrating a stronger inhibitory effect than the ADSC, EXO, MTX, and AE-MTX groups. Additionally, the AE@SiO₂-MTX group showed a marked rise in CD206^+^ cell populations. These findings align with the immunofluorescence results, confirming that AE@SiO₂-MTX nanoparticles can effectively suppress the M1 polarization in macrophages while promoting their transition to the M2 phenotype.

RA-FLS induce synovial tissue and cartilage damage by their migration, invasion, and release of pro-inflammatory factors. It has been reported that ADSC-EXO can significantly promote the differentiation, migration, and proliferation of dermal fibroblasts, consequently accelerating the wound-healing process 47. Nevertheless, its potential therapeutic value as a drug carrier for treating RA remains underexplored.

**Figure 4:**
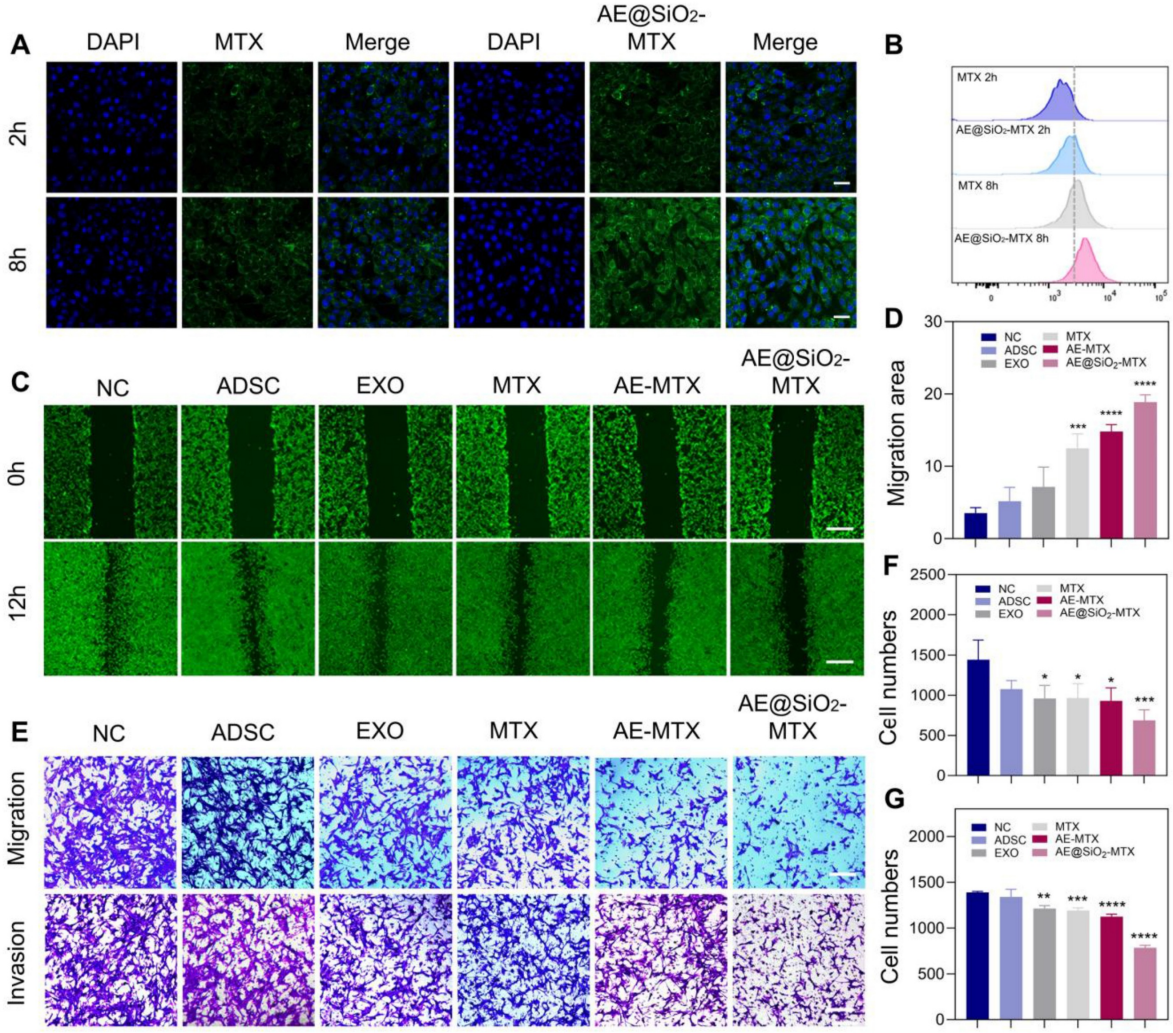
** AE@SiO_2_-MTX inhibited RA-FLS migration and invasion *in vitro*.** (A) and (B) FITC-MTX and FITC-AE@SiO_2_-MTX uptake by FLS in CLSM and flow cytometer. Scale bar: 20 μm. (C-D) Photos and migration areas of FLS 12 h after incubation with PBS, ADSC, EXO, MTX, AE-MTX and AE@SiO_2_-MTX respectively. Scale bar: 100 μm. (E-G) Migration and invasion of FLS 24 h after incubation with PBS, ADSC, EXO, MTX, AE-MTX and AE@SiO_2_-MTX respectively. Scale bar: 100 μm. Data were presented as mean SD. Statistical significance was calculated by one-way ANOVA, n = 3 per group. *P < 0.05, **P < 0.01, ***P < 0.001, ****P < 0.0001.

Before functional evaluation, we co-cultured ADSC (supernatant 200 μL/mL), EXO (2 μg/mL), MTX, AE-MTX, and AE@SiO_2_-MTX (at an equivalent of 1 µg/mL) with FLS to assess cytocompatibility using CCK-8 assays. The findings indicated that the sustained release of MTX resulted in reduced cytotoxicity of AE@SiO_2_-MTX in the test group, ranking second only to the ADSC and EXO groups in terms of safety (Figure S6), which was further supported by the results of the AM and PI staining (Figure S7). CLSM and flow cytometry analyses revealed significant internalization of FITC-AE@SiO_2_-MTX within FLS compared to free FITC-MTX, demonstrating its exceptional biocompatibility (Figure 4A and 4B).

To investigate the potential of AE@SiO_2_-MTX in inhibiting FLS migration and invasion, FLS were incubated with PBS, ADSC, EXO, MTX, AE-MTX, and AE@SiO_2_-MTX in Transwell experiments. Apart from the PBS and ADSC groups, all the experimental groups showed reduced migration and invasion of FLS. However, AE@SiO_2_-MTX exerted a superior blocking effect on FLS compared to the other groups (Figure 4E-G). AM-staining in wound-healing assays confirmed these findings (Figure 4C-D).

**Figure 5:**
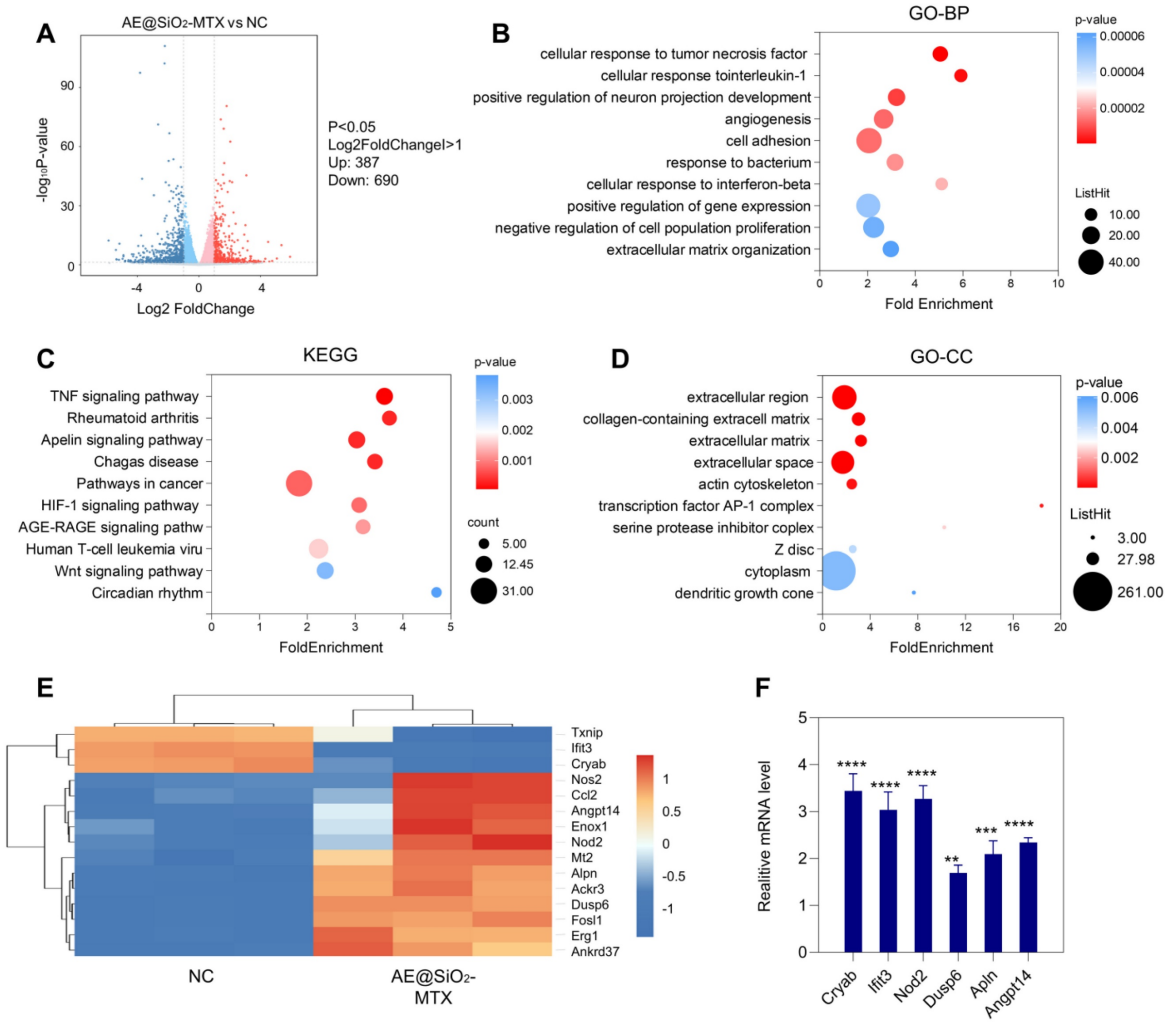
** Metabolic analysis of AE@SiO_2_-MTX in RA-FLS.** (A) Differential gene statistics. (B) and (D) GO and (C) KEGG pathway analyses conducted on the genes of the top 10 mRNAs with the most significant expression in the AE@SiO_2_-MTX group versus NC group. (E) Cluster heatmap of DEGs in AE@SiO_2_-MTX and NC group. BP: biological process; CC: cellular component; Data were presented as mean SD. Statistical significance was calculated by unpaired* t*-test, n = 3 per group. **P < 0.01, ***P < 0.001, ****P < 0.0001.

The mechanism involved in the inhibition of RA-FLS migration and invasion was investigated using RNA sequencing (RNA-seq) of the AE@SiO_2_-MTX and control groups. As shown in the volcano plot, 1077 DEGs were identified between the AE@SiO_2_-MTX and negative control (NC) groups, including 690 downregulated and 387 upregulated genes (Figure 5A). GO enrichment analysis of the DEGs revealed significant enrichment in multiple categories, including the extracellular region and collagen-containing extracellular matrix in the cellular component category, cellular response to TNF and interleukin-1 (IL-1) in the biological process category, and minor groove of adenine-thymine-rich DNA binding in the molecular function category (Figure 5B and 5D, Figure S8). Studies have confirmed the association between RA and altered composition and dysfunction of the synovial extracellular matrix 48. Collagen is essential for maintaining both the stability of the extracellular matrix and the mechanical properties of articular cartilage 49. Furthermore, it has been reported that TNF-α and IL-1β are key players in the pathogenesis of RA 50-52. The findings of this study further support and strengthen these established associations. KEGG analysis identified DEGs enrichment in 10 pathways involved in FLS function during RA development, including the TNF signaling pathway, FLS, and the apelin and HIF signaling pathways (Figure 5C). Heatmap analysis revealed significant differential expression of the first 15 marker genes in a highly enriched pathway (Figure 5E). The expression levels of genes identified *via* RNA-seq were confirmed by qPCR (Figure 5F, Table S2).

To mitigate the risk of embolization associated with live cell injection, we systematically assessed differences in efficacy between ADSC and ADSC-EXO using a mouse model of exogenous antigen-induced AIA. After treatment, the foot volume in the AIA model group continued to increase, while those in the ADSC group gradually reduced. The most significant decrease was observed in the ADSC-EXO group (Figure S9A). The arthritis score of AIA mice remained consistently high, while those in the ADSC group showed slight improvement with significant decreases in the ADSC-EXO group (Figure S9B). Morphological analysis revealed that compared with the AIA group, paw swelling was markedly reduced in both treatment groups, particularly in mice treated with ADSC-EXO (Figure S9C). Thermal imaging further corroborated these findings, demonstrating that AIA mice showed the highest temperature, and that ADSC-EXO treatment was the most effective in restoring them to the normal range (Figure S9C). X-ray evaluation (Figure S9D) indicated that the joint structures of the NC group were intact, while those in the AIA group showed substantial bone destruction, including cortical discontinuity and osteophyte formation. ADSC treatment partially mitigated these changes, while ADSC-EXO treatment led to the restoration of nearly normal cortical structure with only mild distal tibial erosion. HE staining revealed characteristic pathological changes in the joints of AIA model mice, including extensive inflammatory cell infiltration (Figure S9E). ADSC treatment attenuated these abnormalities. Notably, ADSC-EXO administration led to restoration of the synovial architecture with minimal infiltration of inflammatory cells. Safranin O-Fast Green staining further demonstrated severe articular cartilage degradation in AIA mice, with substantial proteoglycan loss and subchondral bone destruction**.** Compared with the ADSC group, the ADSC-EXO group displayed markedly better preservation of both the cartilage matrix and the underlying bone architecture. In conclusion, compared to ADSC, the ADSC-EXO in alleviating inflammation and bone destruction.

**Figure 6:**
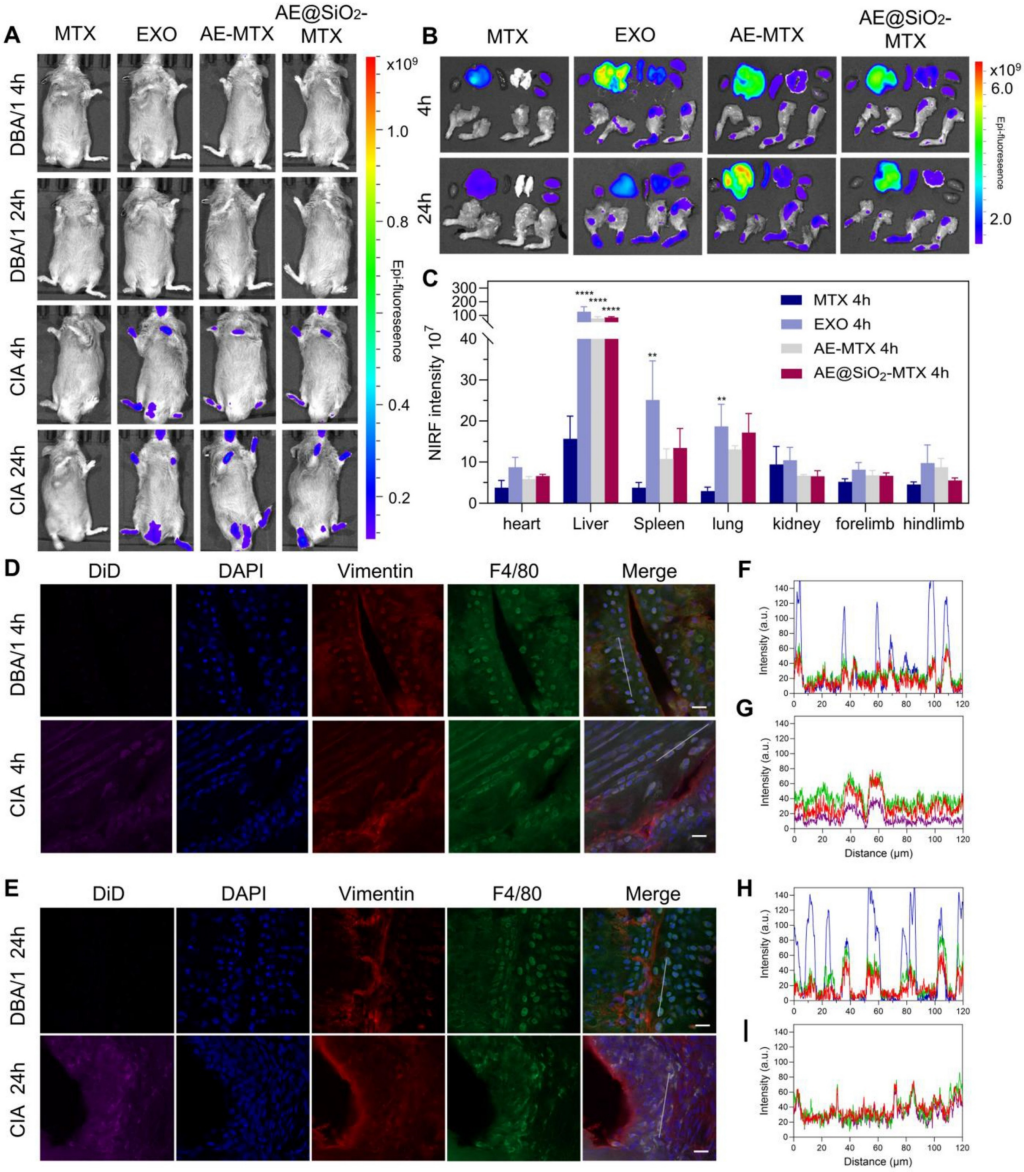
** Distribution of AE@SiO_2_-MTX in CIA mice and cellular presence in joints.** (A) *In vivo* imaging of CIA mice administrated with MTX, EXO, AE-MTX and AE@SiO_2_-MTX at 4 h and 24 h. (B) Images showing fluorescence of major organs and paws from CIA mice after treatments with MTX, EXO, AE-MTX and AE@SiO_2_-MTX at 4 h and 24 h. (C) Measurement of the targeted region, n = 3. (D-E) Frozen joint samples taken from mice post-injection of DiD-labelled AE@SiO_2_-MTX after 4 h and 24 h. purple, DiD; red, fibroblasts (vimentin); green, macrophages (F4/80); blue, DAPI, nuclear staining. Scale bar, 20 μm. (F- I) Co-expression and co-localization of DiD and vimentin and F4/80 were confirmed by line profile analysis. Data were expressed as mean ± SD. Statistical significance was calculated by one-way ANOVA. **P < 0.01, ****P < 0.0001.

Previous studies have shown that MSC exhibit directional migration toward sites of inflammation, a process regulated by specific membrane proteins such as CD29, CD44, and CD73 27-29. Within the vascular system, MSC engage in rolling interactions with the endothelial surface mediated by selectins, with subsequent firm adhesion through interactions with vascular cell adhesion molecules 53-55. Following this, the MSC bind to extracellular matrix components *via* integrins, enabling tissue-specific homing 56. Notably, exosomes secreted by stem cells inherit the functional characteristics of their parent cells. Based on these findings, we further investigated the potential application value of exosomes as a targeted drug delivery system in the CIA model. Cy5-labeled MTX, DiD-labeled EXO, AE-MTX, and AE@SiO_2_-MTX were administered intravenously to CIA mice and their biodistribution was assessed using near-infrared fluorescence (NIRF) imaging. In the control group of normal DBA/1 mice, fluorescence signals were almost undetectable in the paws, indicating that free MTX and DiD-labeled EXO did not accumulate in the joints of healthy mice. Real-time fluorescence imaging in the CIA mice showed that the NIR signals from DiD-labeled EXO, AE-MTX, and AE@SiO_2_-MTX were significantly stronger compared to those in the MTX group, which displayed minimal fluorescence (Figure 6A). The distribution of fluorescent intensity in the joints and extra-articular organs was also assessed (Figure 6B-C and Figure S10). Four hours after the injection, substantial accumulation of EXO, AE-MTX, and AE@SiO_2_-MTX was observed in the joints and paws, contrasting sharply with the MTX group. Even after 24 h, these formulations showed significant retention in the joints and paws, whereas MTX was observed to have accumulated predominantly in the liver and kidneys rather than in the joints or paws. The fluorescence intensities in the heart, spleen, and lung remained relatively low across all treatment groups. These findings demonstrated that EXO maintained the intrinsic targeting characteristics of its parent cells toward sites of inflammation when used as a drug carrier, thus establishing the basis for the nanoparticles to localize rapidly in the inflamed joints.

To further verify the targeting mechanism of AE@SiO_2_-MTX, the relationships between AE@SiO_2_-MTX, macrophages (F4/80), and FLS (vimentin) were assessed using immunofluorescence analysis. The results indicated that AE@SiO_2_-MTX was almost undetectable in normal joints, whereas a markedly increased fluorescence signal was evident in arthritic joints (Figure 6D-E). Fluorescence colocalization analysis further demonstrated a positive correlation between the expression levels of F4/80, vimentin and DiD in CIA mice (Figure 6F-I). These findings collectively indicated that AE@SiO_2_-MTX effectively targeted both FLS and macrophages in the inflammatory micro-environment associated with RA.

**Figure 7:**
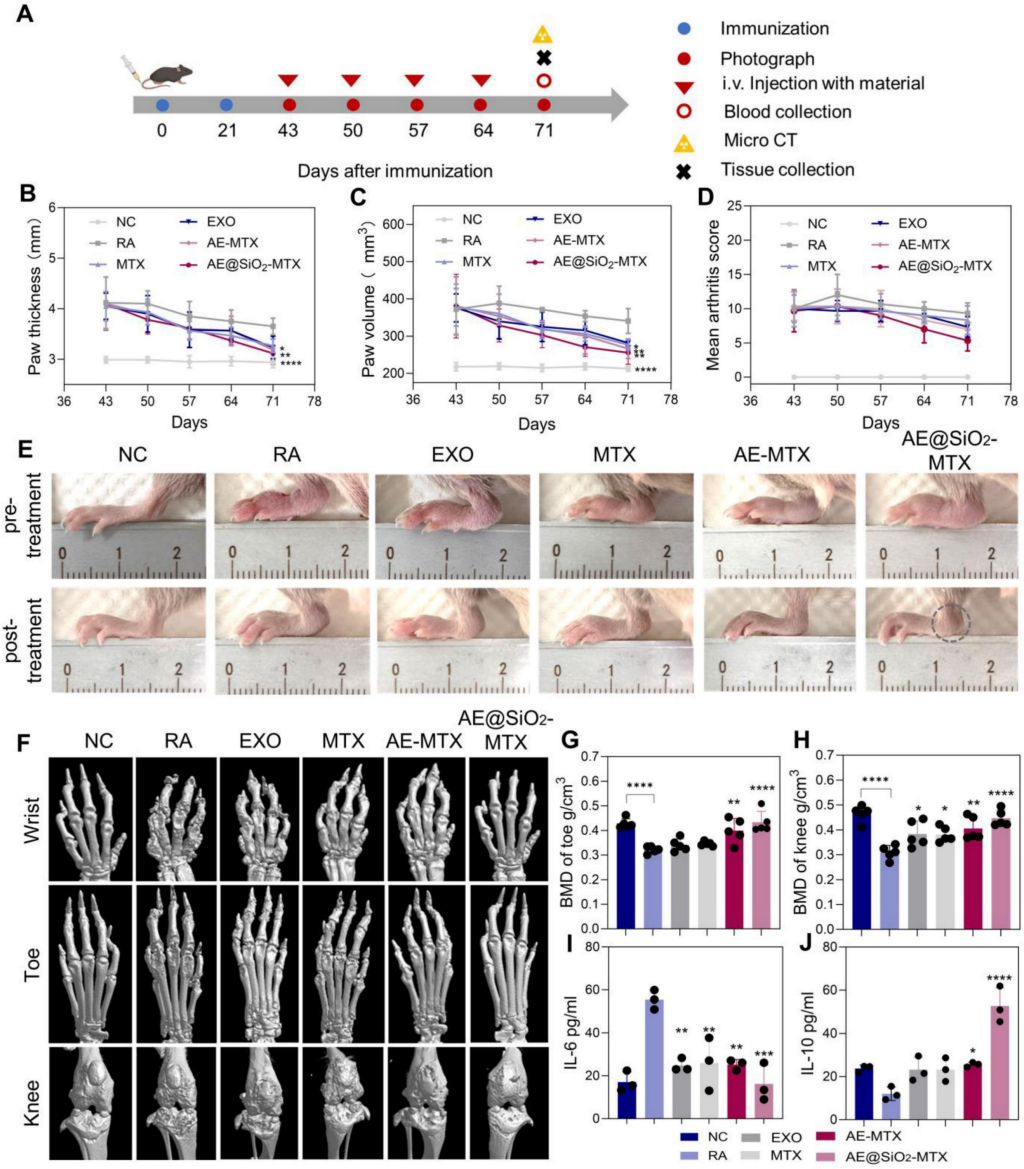
** Intravenous administration of AE@SiO_2_-MTX alleviated arthritis symptoms in CIA mice.** (A) Visual outline of the *in vivo* treatment procedure. (B-D). Quantification of paw thickness, paw volume and arthritis score at multiple intervals after treatments. (E) Illustrative images of hindlimbs from each group before and after treatments. (F) Micro-CT images in 3D of fore paws, hind paws, and knee joints for different treatment groups. (G-H) The evaluation of BMD levels in six groups post-treatments. (I-J) Plasma levels of IL-6 and IL-10 after treatments. Data were expressed as mean±SD. Data were analyzed for statistical significance via One-way ANOVA. *P < 0.05, **P < 0.01, ***P < 0.001, ****P < 0.0001.

We then evaluated the therapeutic impact of AE@SiO_2_-MTX in a CIA mouse model. Schematic representation of the therapeutic procedure in mice with RA is shown in Figure 7A. To investigate its therapeutic properties, MTX was conjugated with SiO_2_ and encapsulated in ADSC-EXO, which were subsequently administered intravenously to mice, while PBS, MTX, EXO, and AE-MTX were used as control treatments. Paw swelling served as a primary measure to assess RA progression. As depicted in the Figure S11, mice treated with PBS showed lower body weight correlating with increased RA severity. The paw joints in the PBS group continued to swell, indicating that PBS alone could not effectively treat RA. Free MTX, EXO, and AE-MTX partly slowed the progression of RA compared to PBS treatment, although none completely inhibited the inflammation in the hind paws. Compared with the other groups, the mice treated with AE@SiO_2_-MTX showed markedly reduced paw thickness and volume, as well as arthritis scores after the final treatments (Figure 7B-D). Representative photographs of hind paws from each group before and after treatments indicated the presence of marked joint redness and swelling before therapeutic intervention in all mice (Figure 7E). The RA group treated with PBS exhibited persistent signs of inflammation, including marked erythema and swelling of the hind paws.

In contrast, both the MTX-treated group and EXO-treated group showed moderate alleviation of joint inflammation, with reduced redness and swelling compared to the control. However, these treatments did not result in complete resolution of inflammatory symptoms. Both the AE-MTX and AE@SiO₂-MTX groups exhibited substantial reductions in joint inflammation. Notably, AE@SiO₂-MTX treatment demonstrated superior anti-inflammatory efficacy, with a more pronounced reduction in ankle swelling (indicated by circles) compared to the AE-MTX group, suggesting greater therapeutic effects.

As paw swelling is only a surface manifestation of RA, it was important to further assess bone erosion by micro-CT analysis in the treated groups. Bone erosion in CIA mice was evaluated using micro-CT imaging, with the results showing varying degrees of bone damage in the wrist, ankle, and knee joints across all treatment groups. Using 3D micro-CT images (Figure 7F) and 2D images (Figure S12) reconstructed in the coronal (COR), sagittal (SAG), and axial (AX) planes, we observed bone erosion in the wrist, ankle, and knee joints of the CIA mice as well as those treated with MTX, EXO, and AE-MTX. Notably, AE@SiO_2_-MTX exhibited superior efficacy compared to the other treatments. Bone repair in CIA mice treated with AE@SiO_2_-MTX was significantly enhanced, with results similar in appearance to those of normal mice. Analysis of BMD indicated a slight improvement in BMD levels among the MTX, EXO, and AE-MTX groups compared to the RA group. In contrast, the AE@SiO_2_-MTX group exhibited BMD levels that approached normal, suggesting significant inhibition against aggressive bone injury (Figure 7G-H, Figure S13).

After CIA model induction, the serum IL-6 levels increased markedly, while those in the free MTX, EXO, and AE-MTX-treated groups decreased substantially following treatment. It was noteworthy that AE@SiO_2_-MTX induced the most pronounced decrease in IL-6 levels. Furthermore, among all treatment groups, the AE@SiO_2_-MTX group showed the most substantial increase in the levels of the anti-inflammatory cytokine IL-10 (Figure 7I-J). Catalase represents a critical enzyme responsible for degrading H₂O₂ to H₂O and O₂, thereby reducing the production of ROS 57. Plasma catalase activity in the AE@SiO_2_-MTX group was significantly higher compared to other treatment groups, suggesting that ROS clearance by the nanomaterials was mediated by upregulation of catalase expression and consequent alleviation of inflammation (Figure S14).

**Figure 8:**
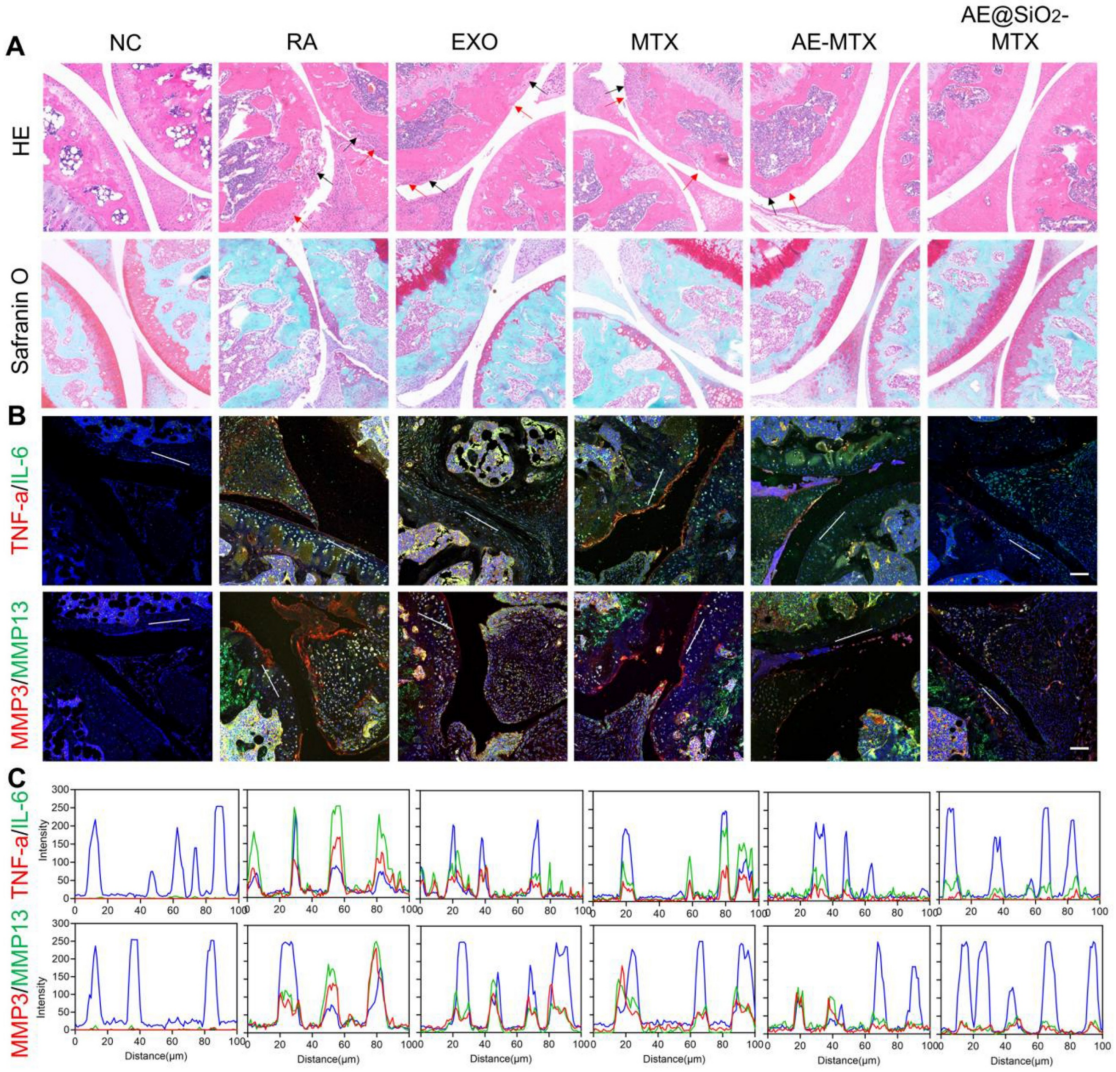
** Therapeutic effectiveness evaluated after administering AE@SiO_2_-MTX.** (A) Knee joints were stained with HE and safranin O. Scale bar: 100 μm. (B) TNF-α, IL-6, MMP3 and MMP13 and DAPI staining applied to knee sections. (C) Line profile analysis verified the co-expression and co-localization of TNF-α and IL-6, MMP3 and MMP13. Scale bar: 50 μm.

In addition to bone erosion, synovitis is also frequently observed in individuals with RA. Following treatments, the knee joints of the mice were subjected to histological staining with HE and Safranin O to evaluate the levels of inflammation and soft tissue disorders. The staining showed varying degrees of leukocyte infiltration (red arrow) and bone destruction (black arrow) in the synovia and cartilage of the RA, MTX, EXO, and AE-MTX groups. However, due to the sustained release of MTX, the AE@SiO_2_-MTX group showed minimal inflammatory changes. Safranin O staining confirmed these findings, demonstrating nearly normal cartilage and bone boundaries in the AE@SiO_2_-MTX group (Figure 8A). In addition, the histological scores of mice treated with AE@SiO_2_-MTX were significantly lower than those in the other groups (Figure S15). These findings indicated that AE@SiO_2_-MTX may have a potential protective effect on synovial tissue and cartilage in the management of RA.

Furthermore, immunofluorescence staining was also conducted on the paw joints, revealing that the levels of TNF-α, IL-6, and MMP-3/13 in the AE@SiO_2_-MTX group were comparable to those observed in healthy mice (Figure 8B, Figure S16-17). These findings were confirmed using fluorescence colocalization (Figure 8C). Therefore, it can be concluded from these findings that AE@SiO_2_-MTX exhibited excellent anti-inflammatory properties in the management of RA.

Histological examination of key organs was conducted to further assess the biocompatibility of the nanomaterial drug delivery. HE staining revealed no notable pathological differences in heart, liver, spleen, lung, and kidney tissues among all groups (Figure S18). The excellent biocompatibility of AE@SiO_2_-MTX may be attributed to its naturally derived peptide composition, facilitating efficient drug uptake and sustained release of MTX for the treatment of RA without inducing adverse effects.

## Conclusion

In this study, we fabricated a bioinspired hierarchical nano-delivery system (AE@SiO₂-MTX) that mimicked native cellular architectures, composed of ADSC-EXO encapsulating SiO_2_-MTX nanocomposites for targeted RA therapy. Compared to free MTX, AE@SiO_2_-MTX demonstrated enhanced pharmacokinetic properties, including sustained drug release kinetics, superior biocompatibility, and rapid cellular internalization. Mechanistically, the system exerted dual therapeutic effects by polarizing pro-inflammatory M1 macrophages toward the anti-inflammatory M2 phenotype and suppressing FLS migration and invasion *in vitro*. Notably, AE@SiO_2_-MTX demonstrated inflammation-responsive joint targeting* in vivo*, significantly alleviating synovial hyperplasia, bone destruction, and cartilage damage in AIA and CIA animal models. The observed immunomodulatory synergy between ADSC-EXO and MTX highlights a paradigm shift toward cell-free nanotherapeutic strategies for RA. Future research directions might include: (1) verification in advanced disease models (e.g., spontaneous arthritis and humanized RA systems) to strengthen clinical translatability; (2) development of bone/cartilage-specific targeting modalities through surface engineering of exosomes; (3) exploration of combinatory payloads to address RA-associated multi-organ comorbidities.

## Supplementary Material

Supplementary figures and tables.
