# Supplementary material for: Bioinspired exosome-SiO2 nanohybrid therapeutic for rheumatoid arthritis treatment
Source: Theranostics. 2025 May 30;15(13):6553–71. doi: 10.7150/thno.108296 (PMC12160022; doi:10.7150/thno.108296)
Supplement: Supplementary file 1 — Supplementary figures and tables. [file thnov15p6553s1.pdf]

Supplementary Information for

Bioinspired exosome-SiO_2 nanohybrid therapeutic for rheumatoid arthritis treatment

Qicui Zhu[1, *], Ruofei Chen[1, *], Xueting Wu[1, *], Yuanyuan Zhou[2], Zexin Wang[1], Huaixuan Zhang[1], Haofang Zhu[1, #], Lingyun Sun [1, 4, #], Zongwen Shuai[1, 3, #]

[1]Department of Rheumatology and Immunology, The First Affiliated Hospital of Anhui Medical University, Hefei, 230022, China

[2]Department of Rheumatology and Immunology, The Second Affiliated Hospital of Anhui Medical University, Hefei, 230601, China

[3]Inflammation and Immune Mediated Diseases Laboratory of Anhui Province, Hefei 230032, China

[4]Department of Rheumatology and Immunology, Institute of Translational Medicine, The Affiliated Drum Tower Hospital of Nanjing University Medical School, Nanjing, 210008, China

[*]These authors contributed equally to this work.

[#]Correspondence:

hfzhu@ahmu.edu.cn, lingyunsun@nju.edu.cn, amushuaizw@163.com

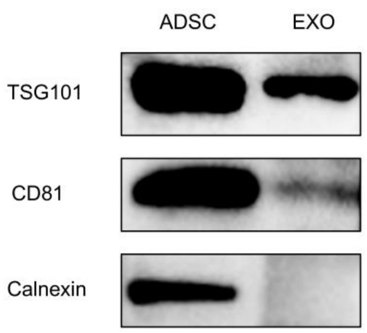

Figure S1. Protein levels of TSG 101, CD81 and Calnexin in ADSC and EXO.

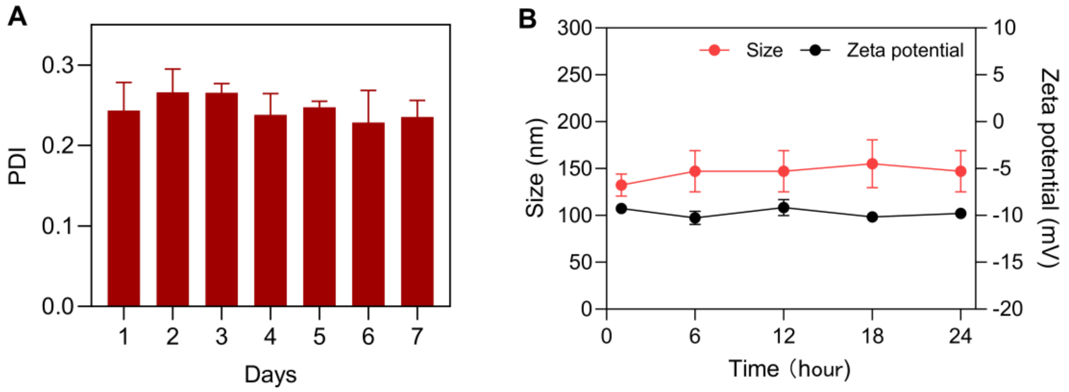

Figure S2. Stability evaluation of AE@SiO_2-MTX nanoparticles: (A) 7-day PDI analysis and (B) serum stability assessment, n = 3 per group.

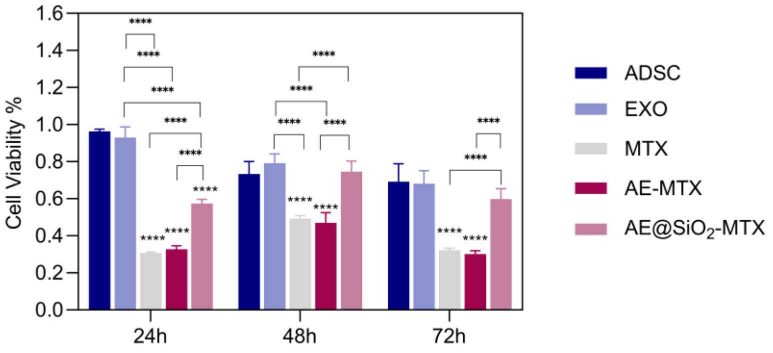

Figure S3. The cytotoxicity of ADSC, EXO, MTX, AE-MTX and AE@SiO_2-MTX to RAW 264.7 cells within 24 h, 48 h and 72 h, n = 3 per group.

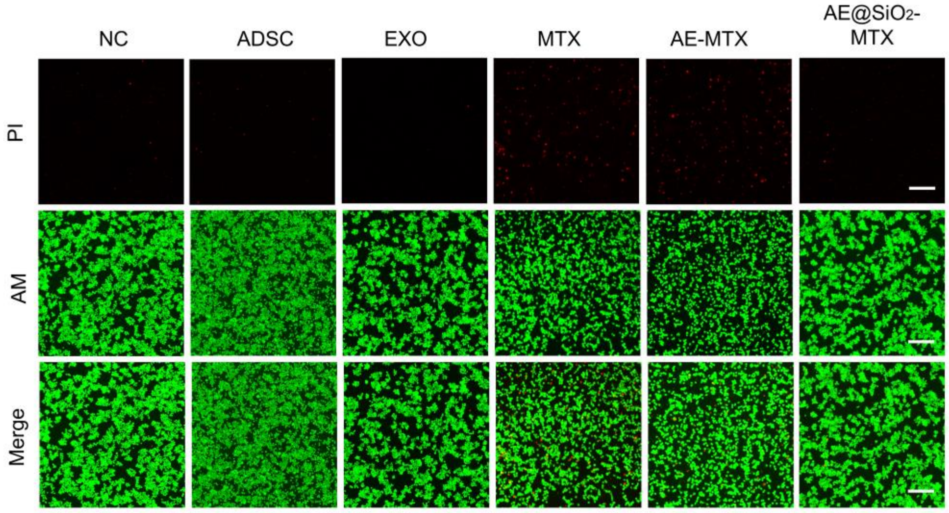

Figure S4. Pictures of RAW 264.7 cells subjected to various treatments. The cells were stained with Calcein-AM (green for live cells) and PI (red for dead cells). Scale bar: 200 µm.

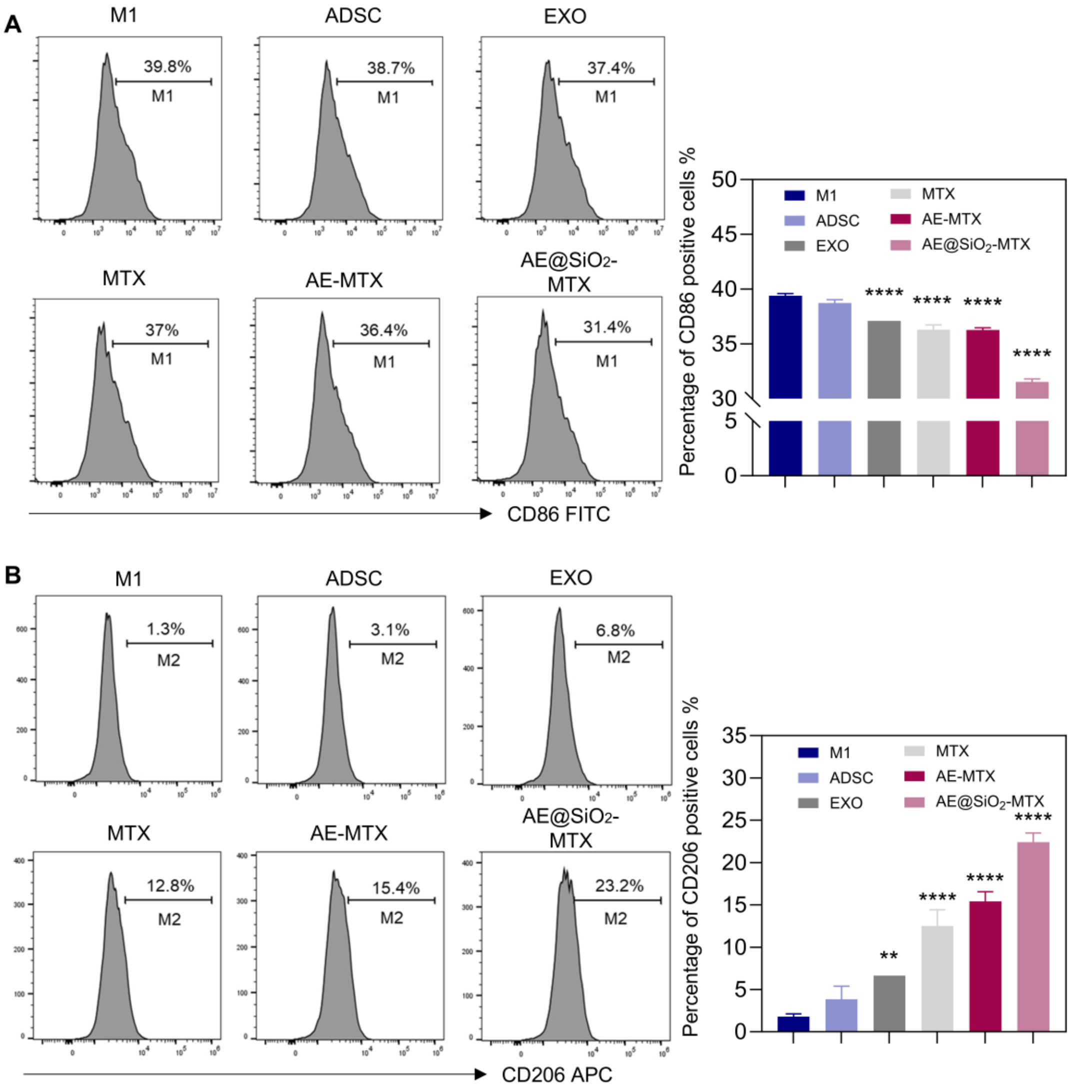

Figure S5. The positive percentages of (A) $CD86^+$ and (B) $CD206^+$ in RAW264.7 macrophages were analyzed by flow cytometry, n = 3 per group.

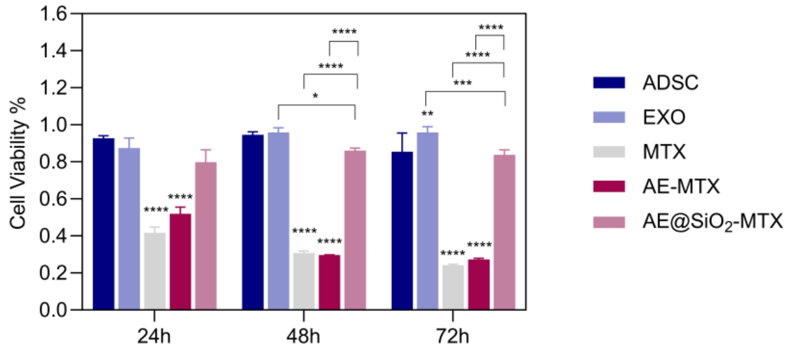

Figure S6. The cytotoxicity of ADSC, EXO, MTX, AE-MTX and AE@SiO_2-MTX to FLS within 24 h, 48 h and 72 h, n = 3 per group.

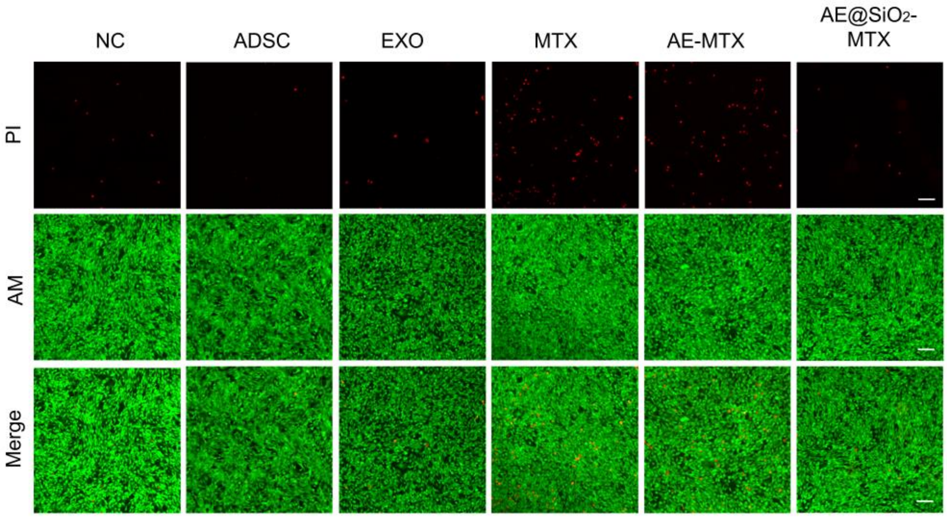

Figure S7. Pictures of FLS subjected to various treatments. The cells were stained with Calcein-AM (green for live cells) and PI (red for dead cells). Scale bar: 200 µm.

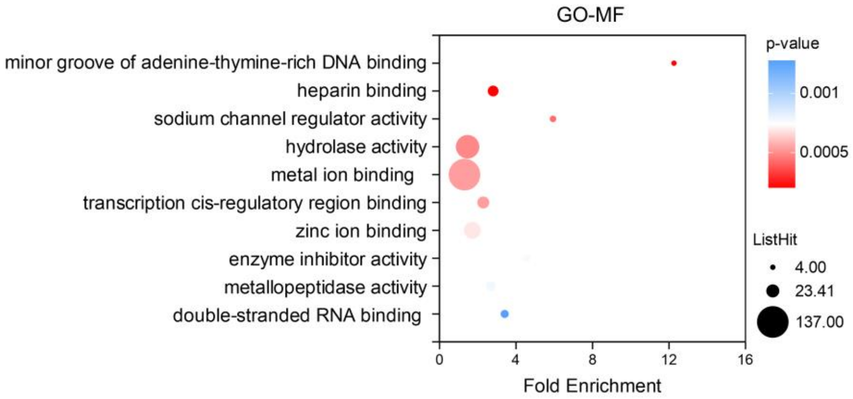

Figure S8. Analysis of GO pathways for target genes of the top 10 significantly expressed mRNA in the AE@SiO_2-MTX group versus the NC group. MF: molecular function.

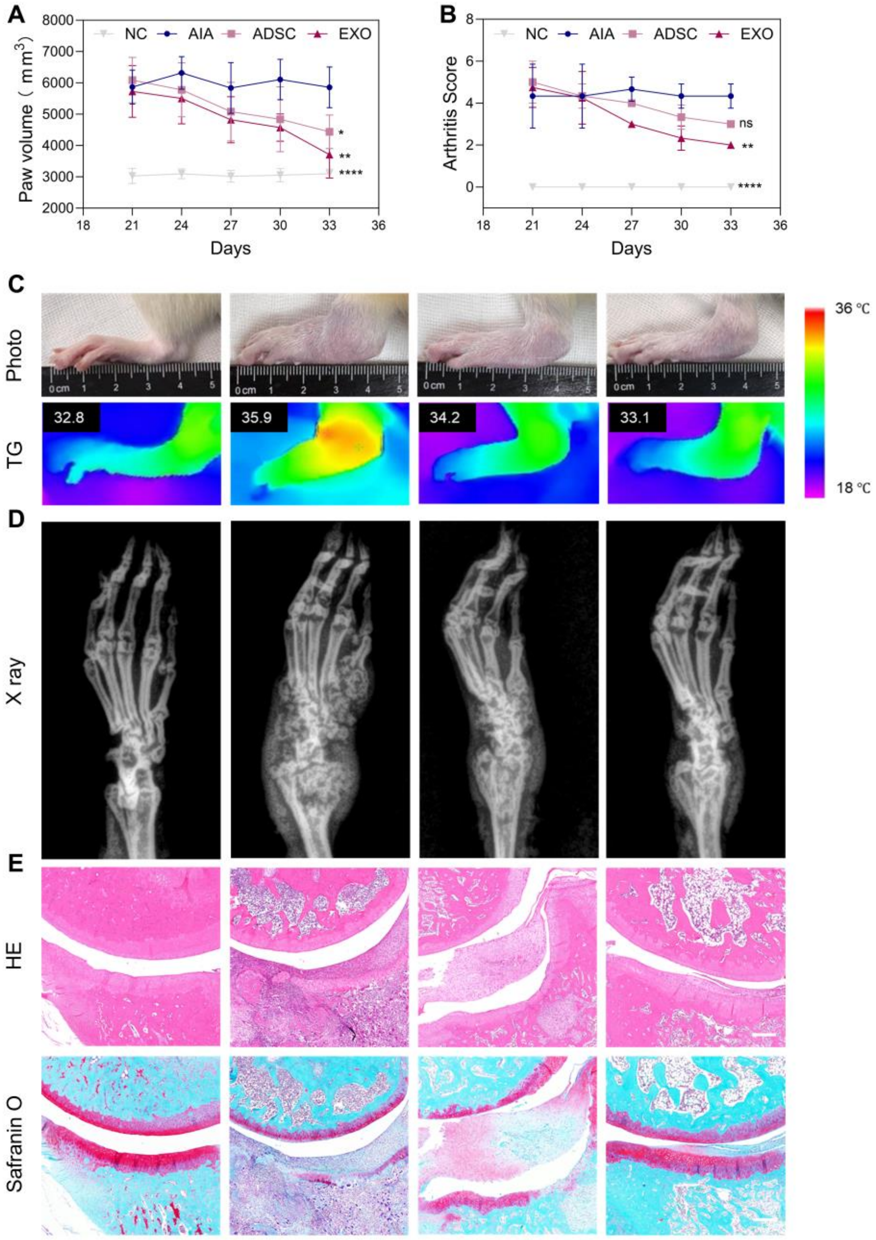

Figure S9. Therapeutic effect of ADSC-EXO on arthritis in AIA rats**.** (A-B) Quantitative analysis of paw volume and arthritis score at different time points after treatment. (C) Representative photographs and thermal imaging of hind limbs post-treatment. (D) Representative X-ray images of each group. (E) Histological analysis by HE and Safranin O staining. Scale bar: 200 μm, n = 3 per group.

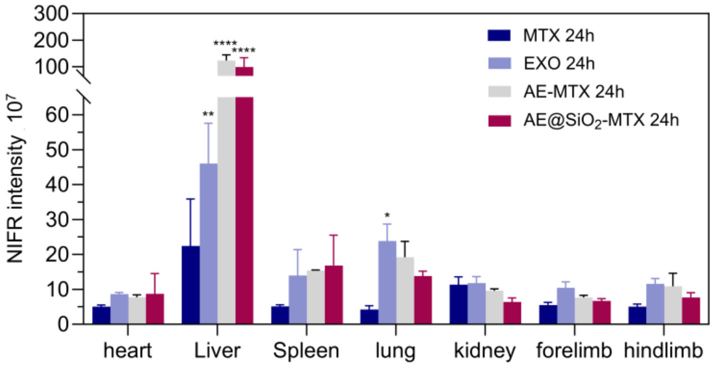

Figure S10. Measurement of the targeted region, n = 3 per group.

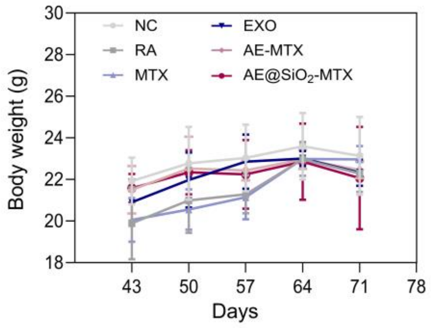

Figure S11. Quantification of body weight at multiple intervals after treatments, n = 3 per group.

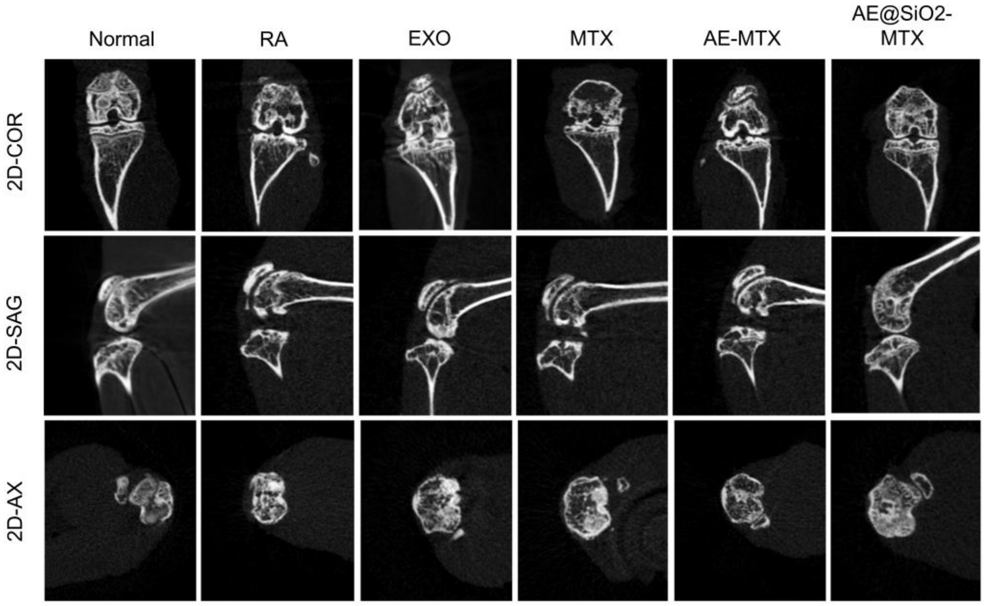

Figure S12. Representative two-dimensional images of the COR, SAG and AX planes.

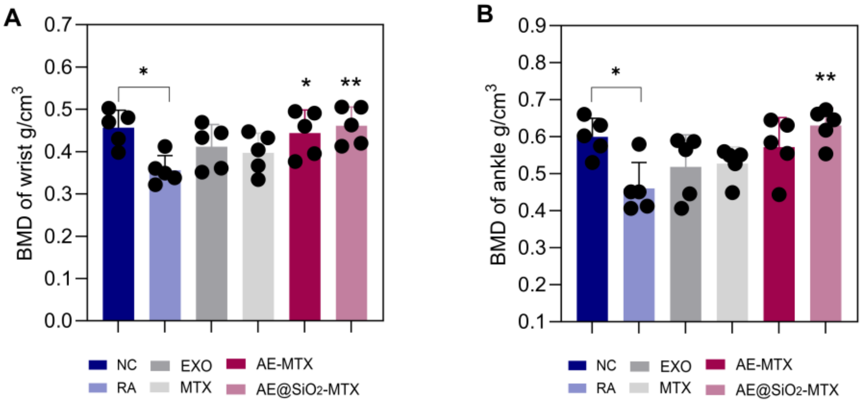

Figure S13. Quantitative analysis of BMD of (A) wrist and (B) ankle, n = 5 per group.

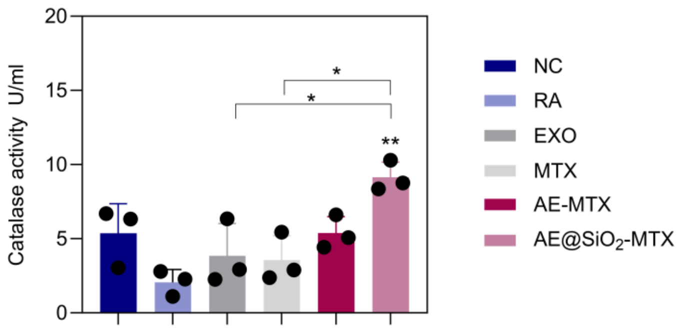

Figure S14. Whole blood levels of catalase activity examined using CAT, n = 3 per group.

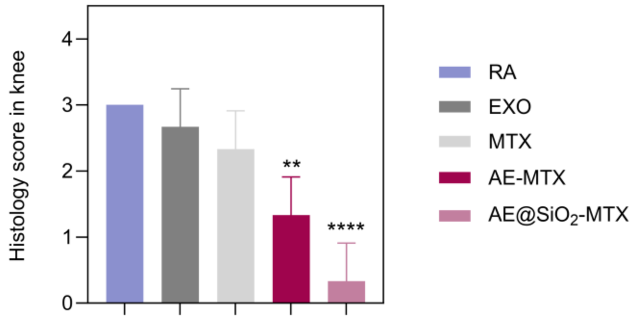

Figure S15. Histological score for knee joints in CIA mice among different groups, n = 3 per group.

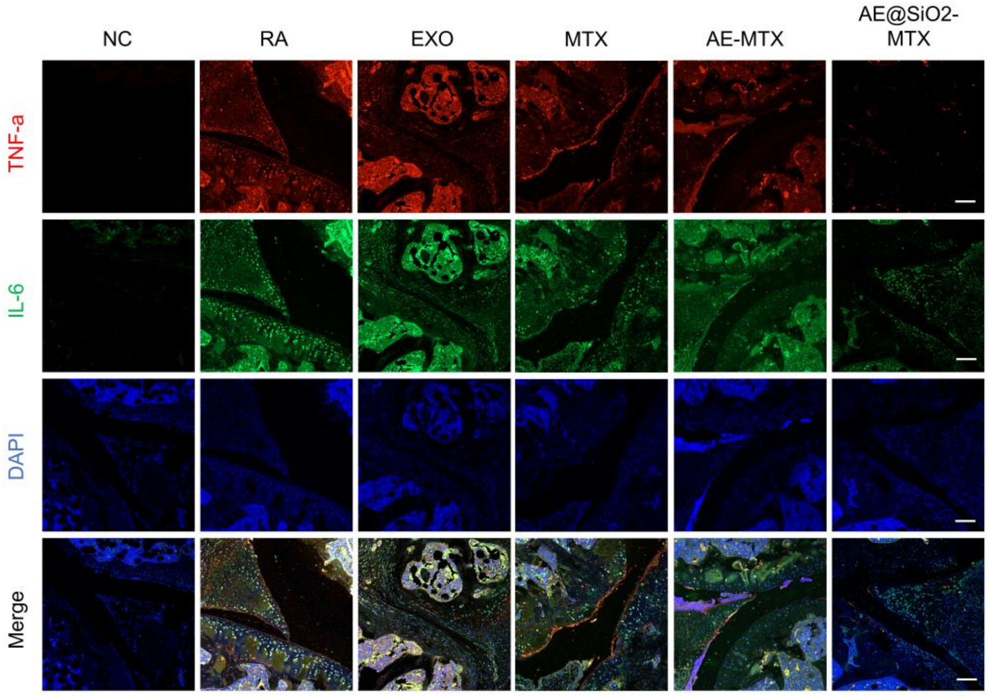

Figure S16. Knee sections immunostained with TNF-α, IL-6, and DAPI, scale bar: 50 μm.

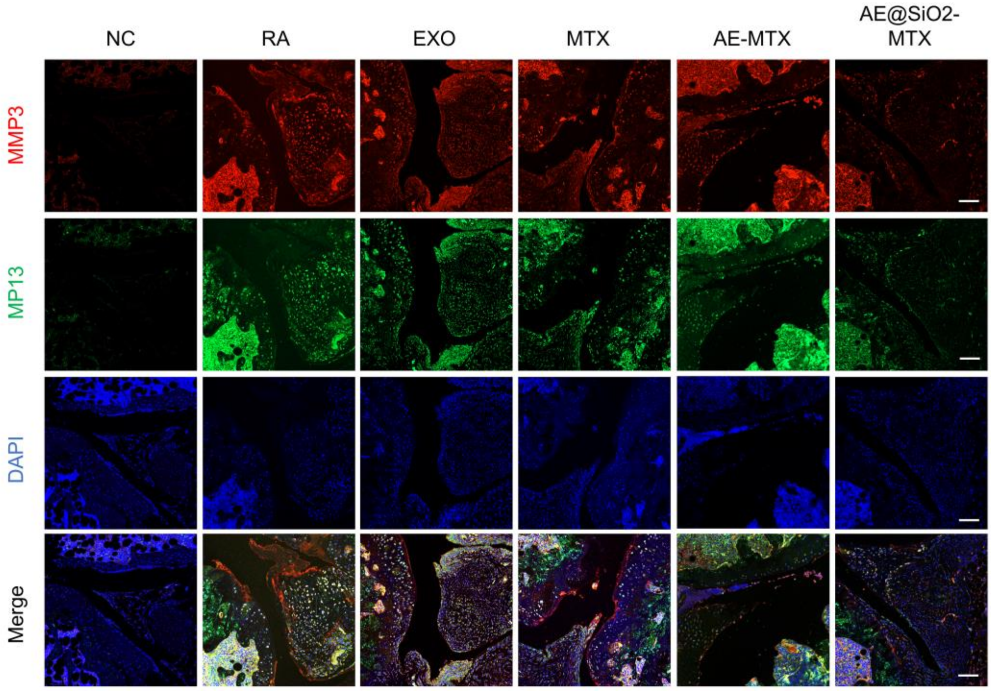

Figure S17. Knee sections immunostained with MMP3, MMP13 and DAPI, scale bar: 50 μm.

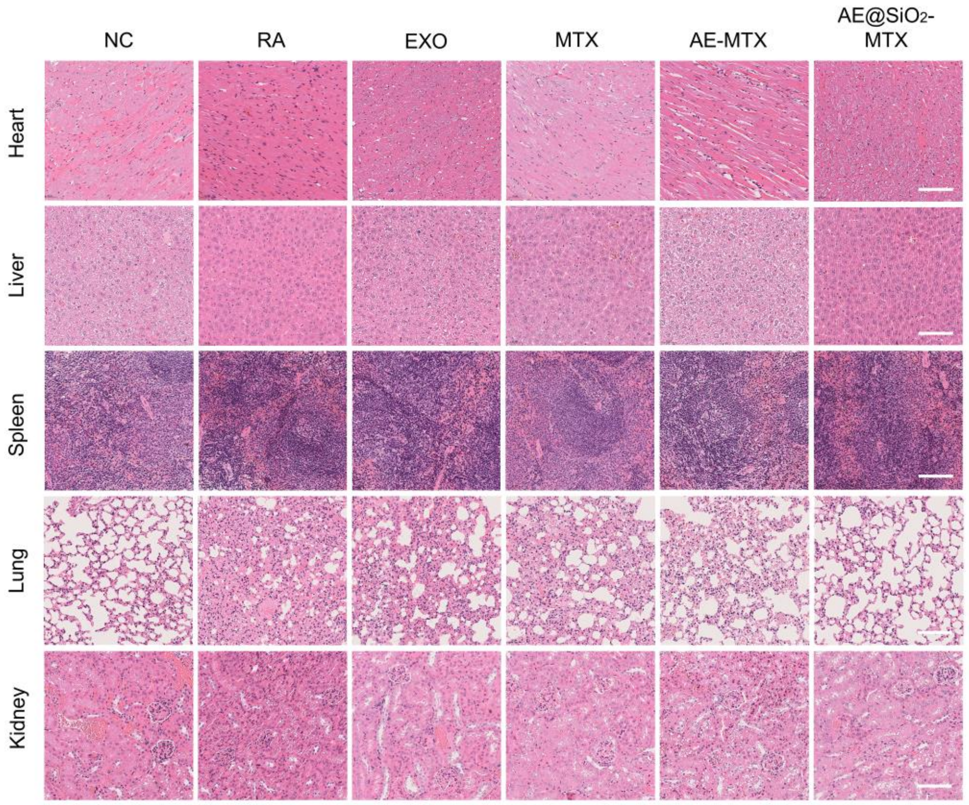

Figure S18. HE staining of heart, liver, spleen, lung and kidney of mice with a scale bar set at 100 μm.

Table S1. EE and DLC of MTX in mesoporous silica with varying proportions.

SiO_2: MTX	EE	DLC
5:1	48%	8%
2:1	64%	24%
1:1	70%	40%

EE: encapsulation efficiency; DLC: drug loading capacity.

Table S2. List of individual gene primers.

Gene	Forward	Reverse
β-actin	GGCTGTATTCCCCTCCATCG	CCAGTTGGTAACAATGCCATGT
Angpt14	CATCCTGGGACGAGATGAACT	TGACAAGCGTTACCACAGGC
Apln	GTGGAGTGCCACTGATGTTG	CTGGTCCAGTCCTCGAAGTT
Dusp6	ATAGATACGCTCAGACCCGTG	ACTAGCAGAAGCCGTTCGTT
Ifit3	CCTACATAAAGCACCTAGATGGC	ATGTGATAGTAGATCCAGGCGT
Cryab	GTTCTTCGGAGAGCACCTGTT	GAGAGTCCGGTGTCAATCCAG
Nod2	CAGGTCTCCGAGAGGGTACTG	GTCACGGATGAGCCAAATGAAG
